# Construction of Reconfigurable and Polymorphic DNA Origami Assemblies with Coiled‐Coil Patches and Patterns

**DOI:** 10.1002/advs.202307257

**Published:** 2024-03-08

**Authors:** Teng Teng, Julio Bernal‐Chanchavac, Nicholas Stephanopoulos, Carlos E. Castro

**Affiliations:** ^1^ Department of Mechanical and Aerospace Engineering The Ohio State University Columbus OH 43210 USA; ^2^ School of Molecular Sciences Arizona State University Tempe AZ 85287 USA; ^3^ Center for Molecular Design and Biomimetics The Biodesign Institute, Arizona State University Tempe AZ 85287 USA

**Keywords:** adaptive materials, coiled coils, DNA origami, DNA‐peptide assemblies, dynamic assemblies

## Abstract

DNA origami nanodevices achieve programmable structure and tunable mechanical and dynamic properties by leveraging the sequence‐specific interactions of nucleic acids. Previous advances have also established DNA origami as a useful building block to make well‐defined micron‐scale structures through hierarchical self‐assembly, but these efforts have largely leveraged the structural features of DNA origami. The tunable dynamic and mechanical properties also provide an opportunity to make assemblies with adaptive structures and properties. Here the integration of DNA origami hinge nanodevices and coiled‐coil peptides are reported into hybrid reconfigurable assemblies. With the same dynamic device and peptide interaction, it is made multiple higher‐order assemblies (i.e., polymorphic assembly) by organizing clusters of peptides into patches or arranging single peptides into patterns on the surfaces of DNA origami to control the relative orientation of devices. The coiled‐coil interactions are used to construct circular and linear assemblies whose structure and mechanical properties can be modulated with DNA‐based reconfiguration. Reconfiguration of linear assemblies leads to micron scale motions and ≈2.5‐10‐fold increase in bending stiffness. The results provide a foundation for stimulus‐responsive hybrid assemblies that can adapt their structure and properties in response to nucleic acid, peptide, protein, or other triggers.

## Graphical abstract

Patterning coiled‐coil interactions on dynamic DNA origami devices enables the controlled formation of hybrid DNA‐peptide reconfigurable assemblies. The arrangement of coiled‐coil peptide interactions specifies assembly pathways to form circular or linear assemblies, and combining coiled‐coil assembly with DNA‐based reconfiguration provides control over assembly structure and properties.

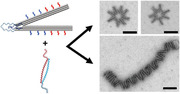

## 1 Introduction

Molecular self‐assembly is a promising route to construct biomimetic or bioinspired materials that leverage the diverse properties and interactions of biomolecules.^[^ ^1^ ^]^ Nature produces many examples of self‐assembled adaptive materials such as actin filaments that form bundles via local interactions with actin‐binding proteins, in order to modulate mechanical properties.^[^ ^2^, ^3^, ^4^ ^]^ Biomolecular nanotechnology provides a useful approach to mimic many of the structural, dynamic, and mechanical features of these systems. In particular, deoxyribonucleic acid (DNA) nanodevices have been designed to exhibit programmed reconfigurations in response to a variety of triggers,^[^ ^5^, ^6^ ^]^ for example to actuate the application of forces to biomolecules.^[^ ^7^ ^]^ Integrating these dynamic device functions into higher‐order self‐assembled architectures could provide an interesting approach to mimic the emergent properties of biomaterials such as adaptive structure and stiffness, large‐scale shape changes, and stimulus‐driven assembly or disassembly.^[^ ^8^, ^9^, ^10^ ^]^ Here we leverage the interaction properties of coiled‐coil peptides and the structural and dynamic properties of DNA origami to make hybrid DNA‐peptide constructs where reconfiguration of the DNA devices can regulate the structure and mechanical properties of higher‐order assemblies.

DNA has emerged as one of the most common materials for the versatile self‐assembly of precise nanostructures and dynamic nanodevices.^[^ ^11^, ^12^, ^13^, ^14^ ^]^ In particular, DNA origami^[^ ^15^, ^16^ ^]^ leverages base‐pairing interactions between a long ssDNA scaffold and hundreds of short ssDNA staples to create nanostructures with complex shapes and programmable motion.^[^ ^17^, ^18^ ^]^ Here we take advantage of these features to construct higher‐order dynamic assemblies. Prior work has established the hierarchical assembly of DNA origami nanostructures to scale up the overall dimensions, demonstrating methods to make micron‐sized assemblies with precisely organized components.^[^ ^19^, ^20^, ^21^ ^]^ Recent examples have further demonstrated building dynamic properties into these higher‐order assemblies; for example: controlling growth/disassembly;^[^ ^22^ ^]^ actuating changes in chirality,^[^ ^23^ ^]^ cross‐section,^[^ ^24^ ^]^ bending,^[^ ^25^ ^]^ or length^[^ ^26^ ^]^ in 1D assemblies; or changing shape in 2D assemblies.^[^ ^27^, ^28^ ^]^ Building on these prior efforts, here we aimed to integrate reconfiguration and assembly to make materials where controlled changes in the conformation of DNA origami devices can control higher‐order assembly structure and mechanical properties.

Furthermore, as a step toward integrating the advantages of DNA‐based assemblies with amino acid materials, we combined the structure and dynamic properties of DNA origami devices with the tunable interactions of peptides.^[^ ^29^, ^30^ ^]^ Peptides have a number of useful properties such as biocompatibility;^[^ ^31^ ^]^ well‐established approaches for sequence design;^[^ ^32^ ^]^ and synthesis,^[^ ^33^ ^]^ specific binding and self‐assembly capabilities,^[^ ^34^ ^]^ and an existing basis for stimulus‐responsive motifs.^[^ ^35^ ^]^ Here, we leverage the specific binding interactions of coiled‐coil peptides. Coiled‐coil interactions,^[^ ^36^ ^]^ which consist of binding between two α‐helical peptides driven by hydrophobic and charge–charge interactions, are one of the most abundant protein assembly motifs found in nature. Previous research has identified peptide sequences that lead to coiled‐coil interactions with well‐understood design principles.^[^ ^33^ ^]^ DNA‐modified coiled‐coils have been demonstrated as a good hybrid adhesive for building higher‐order structures, such as DNA origami filaments,^[^ ^37^, ^38^ ^]^ as well as to integrate DNA nanostructures with functional proteins bearing complementary fused coils.^[^ ^39^ ^]^ Coiled‐coils are also, in principle, reversible through an analogous mechanism to toehold‐mediated strand displacement, through the addition of a fully complementary coil.^[^ ^40^ ^]^

Expanding on this approach, here we employ coiled‐coil interactions to construct assemblies that harness the reconfigurability of dynamic DNA origami devices. We demonstrate the ability to control the conformation of a dynamic DNA origami device with coiled‐coil peptides. In addition, we used coiled‐coil interactions as an adhesive to assemble multiple assembly configurations from the same DNA origami structure design (i.e., polymorphic assembly). To achieve these distinct configurations, we engineered specificity into the coiled‐coil adhesion by patterning two peptides (which form a heterodimeric coiled‐coil) on the DNA origami surfaces to control the relative orientation of adjoining devices, enabling the formation of distinct higher‐order assemblies from the same dynamic DNA origami building block (i.e., polymorphic assembly). Furthermore, we combined coiled‐coil assembly with strand displacement reconfiguration of dynamic DNA origami devices to modulate assembly structure and properties. Taken together, our results demonstrate hybrid DNA origami‐peptide dynamic structures as useful building blocks for assemblies with polymorphic and reconfigurable structures and adaptive properties. Furthermore, the use of coiled‐coil peptides provides an entirely orthogonal interaction mode to DNA base‐pairing. This provides a useful alternative to assemble or reconfigure DNA devices in situations where the addition of large amounts of free DNA may be undesirable, for example in the presence of other charged objects or proteins that might interact with free DNA.^[^ ^41^, ^42^ ^]^ We also found the coiled‐coil assembly helped to mitigate aggregation, which is a common challenge of higher‐order assembly with DNA sticky ends. In the future, this approach can also enable facile integration of functional proteins into the hierarchical nanostructures and allow these nanostructures to respond to protein cues that modulate assembly, conformations, or properties.

## 2 Results

### 2.1 DNA Origami and Peptide Components

**Figure 1:**
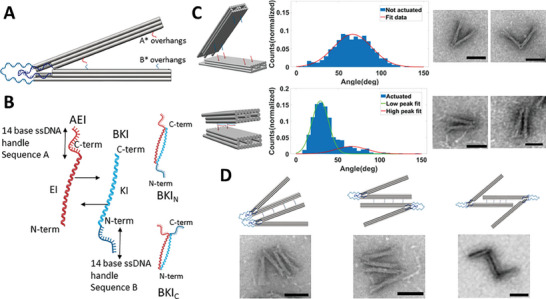
A) Schematic of the DNA origami hinge design consisting of two arms, each a 3 × 8 helix square lattice bundle that is 210 bp long, connected by flexible ssDNA linkers that allow for rotation. The arms can include two groups of ssDNA overhangs for binding coiled‐coil peptides. B) Three DNA‐peptide conjugates were used for reconfiguration and assembly. The AEI conjugate has a 14 nt DNA handle “A” attached to the coiled‐coiled peptide EI on the C‐terminal end. The other conjugate BKI has a 14 nt DNA handle “B” attached to either the C‐terminal (BKI_C_) or N‐terminal (BKI_N_) of the KI peptide conjugate. Combining the AEI and BKI conjugates yields a self‐assembling coiled‐coil interaction where the relative orientation of the handles depends on the use of BKI_C_ or BKI_N_. C) The angular conformations of non‐actuated hinges, depicted schematically (left), were quantified from TEM images (representative single hinges shown at right) revealing a flexible distribution of angles primarily in the range of 50–100 ^o^ (Total of 852 counts, Gaussian fit shown in red). Hinges actuated with coiled‐coil interactions, shown schematically (bottom left), exhibited a large population of constrained angles in the range of 20–40 ^o^ (Total of 670 counts). Red and green lines show individual peaks of a double Gaussian model fit. D) Hinge dimers were assembled via AEI‐BKI conjugates connecting the outer surfaces of the top and bottom hinge arms, leading to a mixture of dimers with hinges in the same orientation (left) or opposing orientations (middle). Locating the AEI‐BKI interactions to the inner arm surfaces leads to dimers with only opposing orientations (right). Scale bars are 50 nm.

We employed a previously developed dynamic DNA origami hinge design with tunable mechanical properties as the basis for our hybrid assemblies.^[^ ^43^ ^]^ The hinge arms are comprised of 20‐helix bundles organized on an 8 × 3 square lattice cross‐section with four internal helices removed from the middle layer (**Figure** **1**A). In addition, there are ≈8‐nm protrusions sticking out past the vertex on the back end of the two arms. The arms are connected by eight ssDNA linkers. Four of these connections are short two nucleotide (nt) linkers that form a rotation axis at the hinge vertex, while the other four are long 70 nt ssDNA linkers used to modulate the hinge properties. Two of the four long ssDNA linkers span across the inner layer near the vertex, and the other two long linkers span across the outer layer of helices at the back end of the protrusion. Previous work has established versatile approaches to modulate the hinge's mechanical properties (i.e., the flexibility and minimum energy angle). For this work we implemented the version referred to as nDFS.A^[^ ^43^ ^]^ (Figure S1, Supporting Information), which exhibits a flexible conformational distribution with most angles falling between 50–100^o^ (Figure S2, Supporting Information).

We also implemented coiled‐coil peptides that were previously utilized in the higher order assembly of static DNA origami nanostructures.^[^ ^37^ ^]^ These peptides consist of 28 amino acid residues consisting of four 7‐residue (heptad) sequences; the peptide with heptad repeat EIAALEK is referred to as referred to as EI, and the peptide with heptad repeat KIAALKE is referred to as KI (Figure 1B). The ends of both EI and KI incorporate azidolysine (azK) residues for conjugation to ssDNA handles by strain‐promoted azide–alkyne cycloaddition (SPAAC) chemistry. These two ssDNA handles, with orthogonal sequences termed A and B, are 14‐nt long and modified with dibenzocyclooctyne (DBCO) at the 5′ end through an amine linker. We followed established protocols^[^ ^37^ ^]^ for the conjugation of ssDNA handle A to peptide EI (conjugate referred to as AEI) and conjugation of ssDNA handle B to peptide KI (conjugate referred to as BKI) (see methods for details). The handle A DNA oligo was conjugated on the C‐terminus of the EI peptide, and handle B was conjugated on either the N‐ or C‐terminus of the KI peptide leading to distinct binding configurations (Figure 1B) with different end‐to‐end distances between the DNA handles. The interaction where the DNA handles are on opposite ends (i.e., A on the C‐terminus of EI and B on the N‐terminus of KI), which we refer to as the N‐terminal configuration, leads to a larger distance between the DNA handles, and the interaction where the handles are on the same end (i.e., A on the C‐terminus of EI and B on C‐terminus of KI) leads to a shorter distance between the DNA handles, which we refer to as the C‐terminal configuration.

### 2.2 Reconfiguration and Assembly of DNA Origami with Coiled Coils

For reconfiguration experiments, we included only one set of overhangs, starting with the set nearer to the vertex. The addition of the AEI and BKI_C_ DNA‐peptide conjugates led to a large shift in the conformational distribution with most hinges exhibiting angles of ≈20–50^o^, as revealed by TEM image analysis (Figure 1C, bottom; Figure S3, Supporting Information), suggesting the coiled‐coil interaction can effectively control the hinge conformation. We performed a mixed two‐Gaussian model fit to the angle distribution data with one population exhibiting an angle of 29 + −10° (i.e., average/peak ± standard deviation from Gaussian fit), corresponding to the actuated hinges, and a second population of hinges exhibiting an angle of 66 + −19°, corresponding to hinges that remained unactuated. Based on these Gaussian fits we estimated a reconfiguration efficiency of 74%. Based on prior work, the coiled‐coil the closed conformation work has shown these small angle conformations can lead to forces of >10 pN applied to interactions between the arms at a similar location and hinge conformation,^[^ ^7^ ^]^ which suggests the efficiency could likely be further increased by adding additional peptide interactions between the arms. We also tested the coiled‐coil reconfiguration in the N‐terminal binding configuration (AEI‐BKI_N_). As expected, these results did not cause a major change in the angle distribution because the larger end‐to‐end distance of the ends of the coiled‐coil attached to DNA would lead to a ≈70^o^ angle. Nevertheless, a slight shift in the angle distribution still suggests some incorporation of the coiled‐coil interaction (Figure S4, Supporting Information). In addition, we also tested designs where the overhangs for binding conjugates were positioned much farther from the hinge vertex (133 bp away) where we observed a larger population of hinges with angular conformations below 50 ^°^ suggesting effective reconfiguration (Figure S5, Supporting Information).

We then studied the use of coiled‐coil interactions to make higher  order assemblies of DNA origami hinge nanodevices. For higher‐order assemblies, we started with dimerization by making multiple sets of hinge devices each with one set of overhangs: one with A* overhangs on the outer face of the top arm, one with A* overhangs on the inner face of the top arm, one with B* overhangs on the outer face of the bottom arm, and one with B* overhangs on the inner face of the bottom arm (Figure S6, Supporting Information). We performed dimerization experiments by combining the outer A* and outer B* devices along with the AEI and BKI_C_ conjugates (we used BKI_C_ conjugates in all assembly experiments), which led to the adhesion of two hinges in multiple possible configurations (Figure 1D, left and middle). Interestingly, we observed 66% of hinge dimers in the same orientation (i.e., vertices pointing in the same direction, Figure 1D, left, Figure S7, Supporting Information). Since the pattern of peptides on the surface is symmetric, either orientation should maximize EI and KI pairing. However, the same orientation dimer configuration might be favored due to slight twisting of the arms, steric interactions of the frayed ends, or possible weak base‐pairing interactions of the ssDNA scaffold loops on the vertex end. We also designed a separate version of hinges where the coiled‐coil interactions adjoin the inner faces of the arms on two separate hinges (Figure 1D, right, Figure S7, Supporting Information).

### 2.3 Polymorphic Assembly of DNA Origami with Coiled Coils

**Figure 2:**
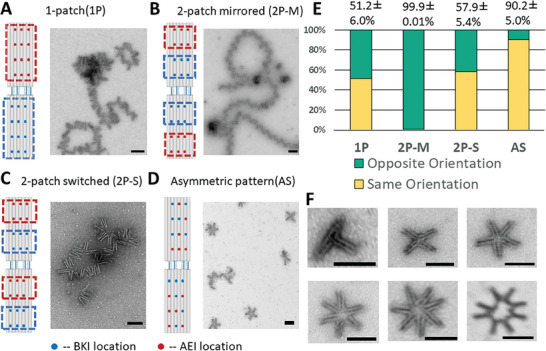
Polymorphic higher‐order self‐assembly with peptide patches and patterns. A) Designing a patch of AEI peptides on the outer surface of the top arm and a patch of B* peptides on the outer surface of the bottom arm leads to higher‐order self‐assembly. The hinges can attach in either orientation leading to assemblies with irregular structure as shown by TEM. B) Designing two patches per arm with BKI nearer the vertex and AEI farther from the vertex yields a controlled assembly of devices in opposing orientations leading to long linear polymers as shown by TEM. C) Switching the arrangement of the patches on the bottom arm again leads to poor control over the orientation as shown by TEM, likely because either arm can attach to either arm. D) Engineering an asymmetric pattern of peptides facilitates the assembly of hinges in similar orientations leading to circular assemblies as shown by TEM. E) The ratio fraction of neighboring devices that assemble in a similar (vertex pointing in the same direction) or opposing (vertex pointing in opposite directions) was quantified from TEM images (Total counts are 1176, 824, 680, and 1231 for the 1P, 2P‐M, 2P‐S, and AS cases, respectively). F) Circular assemblies can form with different numbers of hinges from 3 up to 8 devices as shown by representative TEM images. Scale bars are 100 nm.

Expanding on the capability of coiled‐coil peptides to assemble dynamic devices, we made new designs for the construction of higher‐order multi‐device assemblies using only this specified coiled‐coil peptide pair (AEI‐BKI_C_) and a single hinge design. As a start, we first functionalized the outer side of one arm of the hinge with several A* overhangs and the other arm with B* overhangs, similar to the dimers, but in this case, the AEI‐BKI_C_ complexes could lead to self‐assembly of larger multi‐device assemblies. **Figure** **2**A shows a representative TEM image (additional TEM images in Figure S8, Supporting Information) of the resulting assemblies, which showed having all AEI on the top arm and all BKI on the bottom arm led to a roughly even mix of neighboring devices that had similar orientations (i.e., vertex pointing in the same direction) or opposing orientations (i.e., vertex pointing toward opposing directions). We observed 51 + −6% of assembly interactions led to neighboring devices in the same orientation. While we observed some circular (i.e., several devices in a row with the same orientation) and linear (i.e., several devices in a row with opposing orientations) assemblies, these arrangements only persisted for a few devices, leading to irregular higher‐order structures. These mixed assembly results are likely due to the entire arm being coated with the same peptide, meaning the assembly interactions can randomly orient in either direction while still maximizing the pairing. While the assembly is efficient, there is no control over orientation, and hence no control over the higher‐order assembly structure.

To achieve uniform higher‐order assemblies, we engineered designs that could self‐assemble into either controlled linear (i.e., all opposing orientation assembly interactions) or circular assemblies (i.e., all similar orientation assembly interactions). We took the approach of patterning A* and B* overhangs on the hinge arms to facilitate preferred binding in a specific orientation of neighboring hinges. We started by testing the two arrangements of overhangs shown in Figure 2B,C. Since the linear polymers require all opposing orientations, we arranged a simple pattern with a “patch” of B* overhangs nearer to the vertex and a patch of A* overhangs farther away from the vertex (both patches consist of 3 × 2 arrangement of overhangs) on the outer face of both arms, which we refer to as 2‐patch mirrored (2P‐M). Hence, neighboring hinges would have to be in opposing orientations to form EI‐KI interactions. In this design EI and KI peptides attach to the same arm, but the spacing between where the neighboring EI and KI peptides anchor to the surface is 32 bp along the length of the arms, which is likely a large enough distance to avoid EI‐KI interactions on the same arm surface. Indeed, TEM imaging revealed linear polymers were accurately formed with 99.9% of neighboring hinges exhibiting the correct opposing orientation (Figure 2B, Figure S9, Supporting Information) and long linear assemblies many over a micron in length, with the longest observed consisting of 78 hinge structures (Figure S10, Supporting Information).

Since this approach yielded long polymers, we chose this design to directly compare DNA‐mediated‐assembly with peptide mediated assembly. For this comparison, instead of adding peptides, we added DNA oligonucleotides that contained the A and B sequences directly in one continuous strand to bridge A* and B* overhangs for higher order assembly. Under the same assembly conditions (hinge concentration, incubation conditions, and assembly strand concentration) this DNA sticky‐end approach led to effective assembly, but significantly more aggregation compared to the peptide assembly (Figure S11, Supporting Information). To expand on this comparison, we performed a series of higher order assembly experiments with hinges at lower concentration (1 nm instead of 5 nm) and a variety of different assembly strand concentrations. These results (Figures S12–S22, Supporting Information) showed DNA mediated assembly consistently yielded more aggregation and the coiled‐coil assembly yielded longer unaggregated polymers. At sufficiently high concentrations of assembly strands, both DNA‐mediated and peptide‐mediated assembly were inhibited likely because free DNA strands or free peptides were occupying binding sites. While the assembly conditions could likely be further optimized, these results suggest the use of coiled‐coil peptide interactions for higher‐order assembly provides a useful alternative to DNA‐mediated assembly that can help overcome aggregation, a common challenge of forming large assemblies with purely DNA‐sticky end interactions.

To facilitate circular assemblies, we initially tested a two‐patch design approach where we reversed the pattern longitudinally in one arm of the hinge (i.e., the bottom arm was changed to have the A* patch near the vertex and B* patch away from the vertex). We found this case led to only 58 + −5% of neighboring devices assembling in the same orientation, again leading to irregular higher order assemblies (Figure 2C, Figure S23, Supporting Information). This lack of specificity in assembly likely arises because the top arm can assemble with either the bottom arm of a different structure (leading to the same orientation) or the top arm of that structure (leading to an opposing orientation). To improve control over the same‐orientation assembly, we reasoned that a more asymmetric pattern would avoid these undesired “top arm‐top arm” and “bottom arm‐bottom arm” assembly interactions, leading to higher efficiency in forming circular patterns. Toward this end, we tested the pattern shown in Figure 2D, which we refer to as an asymmetric pattern (AS). TEM imaging revealed this complex pattern yielded drastically improved control over neighboring devices assembled with the same orientation, leading to efficient formation of circular patterns (Figure S24, Supporting Information). In this pattern, EI and KI peptides are closer to each other on the same surface, but the successful assembly suggests interactions between EI and KI on the same arm can be outcompeted by interactions between EI and KI peptides on an opposing arm, likely because all sites accurately align. To further explore this, we performed similar assembly experiments where we extended the length of DNA overhangs that anchor the AEI and BKI constructs by adding 15 thymine nucleotides prior to the A* and B* overhang sequences. In this case, we observed a little higher order assembly (Figure S25, Supporting Information). This suggests that with sufficient flexibility of motion interactions between EI and KI peptides on the same device can prevent higher order assembly; but in the 2P‐M, 2P‐S, and AS designs the intra‐device interactions are sufficiently outcompeted by interactions between opposing arms to yield efficient assembly.

**Figure 3:**
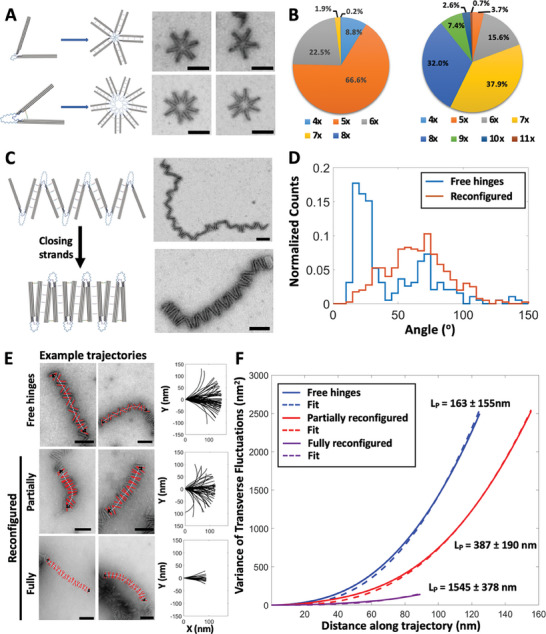
Characterization and control of higher order assemblies. A) Schematic and TEM images of circular assemblies made with free (top) or actuated (bottom) to adopt an angle of 41  ±  13 ^o^ using a DNA strand to form a strut. B) The actuated change to smaller hinge angles led to a shift from predominantly 5 hinges to predominantly 7 or 8 hinges in the circular assemblies. C) Schematic and TEM images of unactuated and actuated linear assemblies where hinges contained DNA overhangs on the inner faces of the arms and DNA closing strands were added after assembling to actuate the polymers. TEM images reveal efficient reconfiguration with some regions appearing straighter than the free hinge polymers. D) TEM image analysis enables quantification of the variation of hinge angle distributions between non‐actuated and actuated polymers. The reconfiguration leads to a population of hinges with an angle of 20 + −2° from 62 + −5 ^o^ (Total counts are 399 and 372 for the free hinge and reconfigured hinge distributions, respectively). E) Examples of trajectory analysis of hinge polymers to evaluate the effects of reconfiguration on the effective persistence length of polymers. The top row is the unactuated hinge polymer. The second row is partially actuated hinge polymer, which means in the trajectories 10 hinges were picked as same as in unactuated ones. The third line is fully actuated hinge polymer, which means in the trajectories only actuated hinges were picked no matter how many hinges assembled together (The total number of trajectories are 40, 63, and 21, for the free hinges, partially reconfigured, and fully reconfigured cases, respectively). F) Analysis of hinge polymer trajectories revealed that the unactuated polymers exhibit an effective persistence length of 163 ±  155 nm. Partially actuated hinge polymers exhibited a persistence length of 387 ±  190 nm, and fully actuated hinge exhibited a persistence length of 1545  ±  378 nm, showing that the reconfiguration leads to an increase in stiffness. Scale bars are 100 nm.

Since the hinge is flexible (i.e., can adopt a range of angles), for circular assemblies of the free hinge were observed containing a varying number of structures. Figure 2F shows examples of circular assemblies containing different numbers of hinge devices (additional TEM images in Figure S24, Supporting Information). The circular assembly containing five hinges was the most abundant at 66%. This is consistent with the average angle of the unactuated hinge, which was ≈70^o^. We then tested our ability to control the number of hinges in circular assemblies by modulating the hinge angle. In order to de‐couple the reconfiguration of hinges from the higher order assembly, we used DNA strands to drive reconfiguration. We introduced a closing DNA strand that actuates the hinge into a 41 ±  13^°^ angular conformation (Figure S26, Supporting Information), and we assembled these actuated hinges into circular patterns using the same asymmetric patterned self‐assembly process. TEM images (**Figure** **3**A, Figure S27, Supporting Information) revealed these constrained hinges led to an increase in the number of devices in the circular assemblies, with the majority of circles containing either 7x (37.9%) or 8x (32.0%) devices (Figure 3B). Although the average hinge angle of 41° might suggest circular assemblies with nine hinges would be likely, we only observed 7.4% of circular assemblies containing nine hinges. This suggests for smaller angles, the average angle does not completely govern the assembly process, likely due to the flexibility of the hinge and the thickness of the arms accounting for additional arc length subtended by each individual hinge. In addition, circular assemblies with a greater number of hinges have a higher probability of a defect (e.g., one opposing orientation assembly interaction), which will inhibit the formation of a fully closed circular assembly.

### 2.4 Adaptive Structure and Mechanical Properties of Hybrid Assemblies

With the circular assemblies, we showed that a priori reconfiguration to modulate the hinge structure can tune the structure of the higher‐order assembly. We next used the linear polymers to demonstrate dynamic control over the structure and properties of the fully formed assemblies. As for circular assemblies, we used DNA to drive reconfiguration to de‐couple the structure change from the higher‐order assembly. We created linear polymers with hinge devices containing DNA overhangs on inner facing sides of the hinge arms, which allow for the closing of the hinge by introducing an additional DNA strand that latches the two arms together by base‐pairing overhangs on each arm (Figure 3C, top). Experiments on isolated hinges showed that this reconfiguration led to hinges with angular conformations of ≈15 + −4 ° (Figure S28, Supporting Information). We observed a similar distribution of angles for hinges incorporated in these linear assemblies compared to isolated free hinges and we found that reconfiguring linear assemblies led to a population of hinges in the polymers shifting from 62 + −5 ° for the free hinges to 20 + −2 ° for the reconfigured hinges (Figure 3D).

In addition to modulating the structure (i.e., changing end‐to‐end distance), the reconfiguration led to assemblies that exhibited relatively straighter regions of the polymers (Figure 3C, Figure S29, Supporting Information). Since not all hinges were reconfigured, the overall shape remains somewhat flexible, but the local decrease in curvature indicates a local increase in stiffness. To quantify this increase in stiffness, we measured an effective polymer persistence length for the linear assemblies with or without reconfiguration. We manually identified and traced the hinges along the polymer, and fit a spline curve to represent the trajectory along the center of the hinges (Figure 3E). We calculated the variance of the transverse fluctuations from these trajectories, which can be correlated to the persistence length as previously described by Isambert et al.^[^ ^44^ ^]^ Fitting the transverse fluctuations of these trajectories resulted in a persistence length of 163 ± 155 nm for the free hinge assemblies (Figure 3F). For the case where we added strands for reconfiguration, we observed incomplete reconfiguration. The angle distribution (Figure 3D) for the hinges in the actuated polymers suggests a reconfiguration efficiency of 72% (using a cutoff of 30 °). The analysis of transverse fluctuations of these trajectories revealed a persistence length of 387 ± 190 nm for the assemblies when we included hinges that remained open in the trajectory analysis (i.e., partially actuated hinge polymers, Figure 3F,E). While these results show a clear increase in stiffness, we hypothesized that the unactuated hinges had a significant impact on the assembly properties. To quantify the properties of fully actuated regions, we traced sections of polymers where all hinges were actuated, which resulted in a persistence length of 1545 ± 378 nm (i.e., full actuated, Figure 3F,E). We interpret this result as indicative of the stiffness over a short distance where hinges are fully actuated, which likely also indicates the limiting behavior if the efficiency were optimized to 100% hinge reconfiguration. To further explore these results, we simulated hinge polymer trajectories using the geometry of the hinges and experimentally measured angle distributions for the free hinge, partially actuated hinge (i.e., full angle distribution for actuated polymers in Figure 3E), and fully actuated hinge (i.e., only selecting angles below 30° from the angle distribution for actuated polymers in Figure 3E). Our simulation results (Figure S30, Supporting Information) yielded persistence lengths of 95, 284, and 5141 nm for the free, partially actuated, and fully actuated polymers. These results are consistent with our experimental results providing further support for the effects of reconfiguration and reconfiguration efficiency on the mechanical properties. Overall, these results illustrate that these dynamic DNA origami‐peptide hybrid assemblies can be actuated to modulate both structure and mechanical properties.

## 3 Conclusion

In this work, we established the use of coiled‐coil peptide interactions in controlling DNA nanodevice conformations and constructing reconfigurable DNA assemblies with dynamic control over structure and mechanical properties. We used the coiled‐coil peptides as an adhesive where the specificity of interactions can be controlled by the location and geometry of peptide interactions. We took advantage of the site‐specific addressability of DNA origami structures to organize individual, patches, or patterns of peptide interactions to direct coiled‐coil interactions for reconfiguration or assembly. These results highlight that even with a single peptide‐peptide interaction (consisting of two complementary coils) we could use spatial organization on the origami to engineer a range of specific multivalent interactions. We demonstrated that isolated coiled‐coil interactions can control device conformation changes, and patches or patterns of peptides can enable controlled higher‐order assemblies. Here we focused on two specific assembly orientations where the hinge arms were fully overlapped and either parallel (same orientation at 0 °) or anti‐parallel (opposite orientation at 180 °). This was achieved by organizing a pattern of peptide interactions with different spacing along the length of the arms versus along the width so the full pattern would only align in the two desired orientations. Then the parallel or anti‐parallel binding was specified by introducing asymmetry into the pattern, with the simplest asymmetry being an arrangement of two patches. For the circular pattern we had to introduce more intricate asymmetry to engineer the specificity of binding between arms, that is the top arm of one device could only bind to the bottom arm (and not the top arm) of another device. This provides an alternative that exploits spatial patterning of interactions instead of sequence design to engineer specificity. Recent advances in the colloidal or patchy particle assembly of DNA origami structures^[^ ^45^, ^46^ ^]^ could provide further guidance toward more complex and functional assemblies.

Interestingly, our studies with linear assemblies suggest using coiled‐coil interactions for higher‐order DNA origami materials assembly can mitigate non‐specific aggregation, which is a common challenge when using DNA sticky ends for higher‐order assembly. This could be particularly useful when many different stands are needed for reconfiguration or the addition of many DNA strands may be undesirable due to the presence of other charged objects,^[^ ^42^ ^]^ electric fields,^[^ ^12^ ^]^ or DNA‐protein interactions.^[^ ^41^ ^]^ We used orthogonal interactions with peptides mediating assembly and DNA mediating conformation changes to achieve either “reconfiguration‐then‐assembly” to modulate the structure of self‐limiting circular assemblies, or “assembly‐then‐reconfiguration” to modulate the structure and mechanical properties of linear assemblies. Prior work has demonstrated DNA origami assemblies with variable mechanical properties;^[^ ^47^, ^48^ ^]^ however, these were not actuated systems, but rather different folded versions of structures that combined into higher‐order assemblies. Here, we showed a 2–3‐fold increase in linear polymer bending stiffness with reconfiguration, with local increases in stiffness of 10‐fold, which could be extended to the full polymer with further optimization.

Our results build on recent efforts to make controllable assemblies of nanostructures.^[^ ^49^, ^50^ ^]^ Many studies have demonstrated higher‐order assembly of static DNA origami structures,^[^ ^19^, ^20^, ^51^ ^]^ and recent studies have extended to dynamic assemblies.^[^ ^22^, ^23^, ^24^, ^25^, ^26^, ^27^ ^]^ These studies have scaled the geometric precision and reconfigurability of DNA origami to establish useful features in higher‐order assembly including the precise control of larger structures through geometric design and self‐limiting assemblies, the ability to form multiple distinct assembly structures using similar building blocks (i.e., polymorphic assembly), and the ability to reconfigure assemblies. Here we focused on combining these features to make assemblies that can be both polymorphic and reconfigurable. We also demonstrated that reconfigurability can not only adapt structure, but also adapt the assembly mechanical properties. The adaptive nature of our assemblies and the combination of peptide and nucleic acid interactions to achieve assembly and reconfiguration suggest these dynamic assemblies could serve as a useful platform to design adaptive materials that change shape or properties in response to amino acid or nucleic acid‐based triggers or other environment stimuli. In particular, the use of coiled‐coils could be an attractive approach for interfacing with a variety of proteins.^[^ ^39^ ^]^

## 4 Experimental Section

### DNA Origami Folding and Purification

The DNA origami hinge was designed as a combination of an 8064 nt ssDNA scaffold^[^ ^50^ ^]^ and staple strands by using software caDNAno.^[^ ^52^ ^]^ The design diagram from caDNAno^[^ ^51^ ^]^ is included in Figure S1 (Supporting Information), illustrating all design details, and the sequences for all staples are included in Table S1 (Supporting Information). Briefly, the parts of overhangs used for binding coiled‐coil peptide conjugates are fully complementary to the DNA handles in conjugates. For AEI peptides, the overhang A* sequence is GTAATACCAGATGG and for BKI peptides, the overhang B* sequence is TATATGGTCAACTG. In addition, overhangs on the inner side of arms in self‐assembly design are used for binding close strands to actuate the structures. The closing strand sequence is AGTGGACCAGTGGGTCTTCGTATAGACCCGACTTTGGGCCTAAGTGGGTCCACACGCACG. The scaffold was prepared as previously described,^[^ ^16^ ^]^ and the staple strands were ordered from a commercial vendor (IDT, Coralville).

Folding reactions contained 20 nm scaffold and 200 nm of each staple strand in a ddH_2_O solution containing 1 ×  FOB and 20 mm MgCl_2_.This folding reaction was subjected to thermal annealing in a thermal cycler (Bio‐Rad, Hercules, CA) consisting of rapidly heating the solution to 70 °C for 15 min, followed by annealing over the range of 63–57 °C for 3 h per degree Celsius, and then cooling for 30 min at 4 °C.

The structures were purified by centrifugation in a polyethylene glycol (PEG) solution.^[^ ^52^ ^]^ The production of folding reaction with folded DNA origami structures was mixed with an equal volume of PEG buffer (15% PEG MW8000, 200 mm NaCl, and 100 mm Tris). The mixture was then centrifuged at 4 °C and 16 000 g for 30 min. The supernatant was removed, and the origami structures were resuspended in 1 ×  FOB with 20 mm MgCl_2_. Then the concentration of the structure in the solution was measured by Nanodrop (NanoDrop 2000C Spectrophotometer, Thermo Scientific) in 260 nm absorbance.

### Reconfiguration

Purified individual structures with group 1 or group 2 overhangs were mixed with 20× excess AEI and BKI conjugates relative to the structure concentrations, and then the final concentration of structures was adjusted to 5 nm with buffer containing 1 ×  FOB and 20 mm MgCl_2_ and final volume is 50 µL. The mixture was subjected to an incubation of 15 h at 37 °C.

### TEM and Analysis

The sample was diluted to 1 nm in 0.5 × TBE with 10 mm MgCl_2_ buffer for transmission electron microscopy (TEM). For TEM grid preparation, 4 µL of sample volume was deposited on Formvar‐coated copper TEM grids, stabilized with evaporated carbon film (Ted Pella; Redding, CA). Then the sample was incubated on the grid for 4 min when incubating a single structure, and for 8 min when incubating self‐assembly structures, and then was wicked away with filter paper. The sample was then stained with 2% uranyl formate (SPI, West Chester, PA, USA). First, a 10 µL drop was applied for 2 s and wicked away to wash the sample, and then another 10 µL drop was applied for 15 s and then wicked away with filter paper. TEM imaging was performed at the OSU Campus Microscopy and Imaging Facility on an FEI Tecnai G2 Spirit TEM using an acceleration voltage of 80 kV at different magnifications.

The raw TEM figures were organized into a gallery containing clear formations (Figures S2–S6, S14–S16, Supporting Information) by using the particle picking tool in the software EMAN2. Then the angles were measured in the software ImageJ by drawing two straight lines directly on each particle along the inner side of each arm in one hinge.

The MATLAB was used as the postprocessing tool to convert the angle data sets to probability density histograms. To estimate the fraction of the hinge in non‐actuated or actuated states, it was used the peakfit MATLAB function program, which used a non‐linear optimization algorithm to decompose a complex, overlapping‐peak signal into its parts, by assuming the conformational distribution consisting of two populations. The peak number was set as two for the actuated sample and the distribution was fit with a combination of two Gaussian distributions, termed as the actuated and non‐actuated parts.

### Self‐Assembly

Purified individual structures with each designed overhang arrangement were mixed with 20× excess AEI and BKI conjugates relative to the structure concentrations, and then the final concentration of structures was adjusted to 5 nm with buffer containing 1× FOB and 20 mm MgCl_2_ and the final volume was 50 µL. For the linear self‐assembly polymer, the mixtures were then subjected to a 1‐cycle low‐temperature annealing ramp starting at 45 °C followed by an annealing phase at −2 h per °C until 20 °C, to prevent extensive aggregation. For other self‐assembly structures, the process was a 2‐cycle annealing ramp, which was repeated twice.

### Circle Pattern Reconfiguration

Before the self‐assembly process, the purified structures were mixed with 0.5 µL of 10 µm closing strands and then incubated at 37 °C for 15 h to yield an angle of hinges mostly actuated to 45 °.

### Linear Polymer Reconfiguration

After the self‐assembly process, 0.5 µL of 10 µm closing strands were added to the production. Then the mixture was incubated to 37 °C for 15 h.

### Persistence Length from Shape Variance in TEM Images

TEM images of non‐actuated and actuated self‐assembly polymers were analyzed using MATLAB. To discretize the shape of the polymers, 11 points on the connection of the hinge along the trajectory were manually selected by clicking on the image Figure 3E. These selected points were used to fit a cubic spline of the trajectory coordinates along the filament path to obtain fine resolution of the curvature. Configurational distributions were obtained by aligning the filament trajectories so that they started at the origin and initially pointed in the horizontal direction Figure 3E and Figure S30 (Supporting Information). Isambert et al. derived a relation between the filament persistence length (LP) and the average transverse fluctuations, 〈[*D*(*s*)]^2^〉,

(1) $$\langle {\left[D\left(s\right)\right]}^{2}\rangle =L_{p}^{2}\;\left[2\frac{s}{L_{p}}+\frac{16}{3}\exp\left(-\frac{s}{2L_{p}}\right)-\frac{1}{3}\exp\left(-\frac{2s}{L_{p}}\right)-5\right]$$

The average transverse fluctuations were determined as a function of arc length from the filament configurational distributions. The *L*_P_ of the actuated and non‐actuated self‐assembly polymer were characterized respectively by calculating the average of the transverse fluctuations squared from the configurational distributions and fitting Equation (1).

### Materials and Supplies for Peptide Synthesis and Conjugation

Fmoc‐protected amino acids for peptide synthesis were purchased from EMD Millipore. Fmoc‐azidolysine was purchased from Combi‐Blocks Inc. Dichloromethane (DCM) was purchased from Millipore Sigma. Dimethylformamide (DMF) and diethyl ether were purchased from Oakwood Chemical Inc. Piperidine was purchased from Alfa Aesar. Oxyma and diisopropyl carbodiimide (DIC) were purchased from ChemImpex. TFA was purchased from Oakwood, and Rink Amide resin was purchased from Novabiochem. Dibenzocyclooctyne‐Sulfo‐N‐hydroxysuccinimide (DBCO‐sulfo‐NHS) linker was purchased from Click Chemistry Tools. All oligonucleotides were purchased from Integrated DNA Technologies.

### Peptide Synthesis and Characterization

Peptides were obtained using solid phase peptide synthesis (SPPS) on a CEM Liberty Blue instrument. Synthesis was performed on a solid phase Rink‐Amide resin (0.78 mmol g^−1^) at a 0.1 mmol scale, using a standard Fmoc protocol and deprotected in 20% piperidine in DMF. Amino acids, coupling agents, DIC, and Oxyma, were added in a 10‐fold molar excess. Crude peptides were cleaved by shaking the resin in a solution containing trifluoroacetic acid (TFA), triisopropylsilane (TIPS), and water in a ratio of 95:2.5:2.5 for 3.5 h. The resin was washed with TFA and concentrated under nitrogen. The solution was then added to 40 mL of cold diethyl ether to precipitate the peptide. The solution was centrifuged at 4200 rpm for 10 min, the supernatant was removed, and the pellet was allowed to dry overnight. The dried pellet was dissolved in a mixture of water and acetonitrile (50:50) and 0.1% TFA. Peptides were purified via reverse phase chromatography on a Waters HPLC using a Phenomenex column with C18 resin. A linear gradient was generated using water/acetonitrile + 0.1% TFA, from 10% to 100% acetonitrile over 50 min. Peak fractions were collected based upon their absorbance at 230 nm and tested for purity by MALDI‐TOF mass spectrometry on a Bruker Microflex LRF MALDI using α‐cyano‐4‐hydroxycinnamic acid matrix (Sigma). Pure fractions were pooled and lyophilized, and peptides were stored at −20 °C until use.

### Synthesis of Peptide‐DNA Conjugates

DNA‐peptide conjugates were prepared via strain‐promoted azide‐alkyne cycloaddition (SPAAC) following reported protocols.^[^ ^37^ ^]^ Briefly, amine modified oligonucleotides were dissolved in phosphate buffered saline (PBS) to a concentration of 1 mm. A 10 molar excess of DBCO‐sulfo‐NHS dissolved in dimethylsulfoxide (DMSO) was then added to the DNA and agitated at RT overnight. The reaction mixture was washed six times with a 3 kDa molecular weight cutoff (MWCO) filter (Amicon) to remove any excess DBCO. The resulting DNA was purified by reverse phase High‐Performance Liquid Chromatography (HPLC) on an Agilent 1220 Infinity LC HPLC with a Zorbax Eclipse XDBC18 column. A Linear gradient was generated using 50 mm TEAA/Methanol from 10% to 100% methanol over 60 min. Peak fractions were collected based upon their absorbance at 260 nm and tested for purity by MALDI‐TOF mass spectrometry on a Bruker Microflex LRF MALDI in 3‐Hydropicolinic acid (HPA) matrix (Sigma). Pure fractions were pooled, and the buffer was exchanged into the water using a 3 kDa MWCO filter. The purified DBCO‐DNA was then mixed with the azido‐peptides, heated to 37 °C, and shaken overnight. Following the reaction, samples were exchanged into water using a 3 kDa MWCO filter, and purified by reverse phase HPLC, similar to the modified DBCO‐DNA.

## Conflict of Interest

The authors declare no conflict of interest.

## Supporting information

Supporting Information

## Data Availability

The data that support the findings of this study are available from the corresponding author upon reasonable request.
