# Supplementary material for: Construction of Reconfigurable and Polymorphic DNA Origami Assemblies with Coiled‐Coil Patches and Patterns
Source: Adv Sci (Weinh). 2024 Mar 8;11(20):2307257. doi: 10.1002/advs.202307257 (PMC11132032; doi:10.1002/advs.202307257)
Supplement: Supplementary file 1 — Supporting Information [file ADVS-11-2307257-s001.pdf]

ADVANCED SCIENCE
Open Access

Supporting Information

for *Adv. Sci.*, DOI 10.1002/advs.202307257

Construction of Reconfigurable and Polymorphic DNA Origami Assemblies with Coiled-Coil Patches and Patterns

*Teng Teng, Julio Bernal-Chanchavac, Nicholas Stephanopoulos and Carlos E. Castro**

Materials and Methods

DNA origami folding and purification

The DNA origami hinge was designed as combination of an 8064 nt ssDNA scaffold[1]and staple strands by using software caDNAno[2]. The design diagram from caDNAno is included in Supplementary Figure S1, illustrating all design details and the sequences for all staples are included in Table S1. Briefly, the parts of overhangs used for binding coiled-coil peptides conjugates are fully complementary to the DNA handles in conjugates. For AEI peptides, the overhang A* sequence is GTAATACCAGATGG and for BKI peptides, the overhang B* sequence is TATATGGTCAACTG. In addition, overhangs on the inner side of arms in self-assembly design are used for binding close strands to actuate the structures. The closing strand sequence is AGTGGACCAGTGGGTCTTCGTATAGACCCGACTTTGGGCCTAAGTGGGTCCACACGCACG. The scaffold was prepared in our laboratory[ref], and the staple strands were ordered from a commercial vendor (IDT, Coralville).

Folding reactions contained 20 nM scaffold and 200 nM of each staple strand in a ddH2O solution containing 1× FOB and 20 mM MgCl2.This folding reaction was subjected to thermal annealing in a thermal cycler (Bio-Rad, Hercules, CA) consisting of rapidly heating the solution to 70°C for 15 min, followed by annealing over the range of 63–57°C for 3 hours per degree Celsius, and then cooling for 30 min at 4°C.

The structures were purified by centrifugation in a polyethylene glycol (PEG) solution [3]. The production of folding reaction with folded DNA origami structures was mixed by an equal volume of PEG buffer (15% PEG MW8000, 200 mM NaCl and 100 mM Tris). The mixture was then centrifuged at 4 °C and 16000g for 30 min. The supernatant was removed, and the origami structures were resuspended in 1× FOB with 20 mM MgCl2. Then the concentration of the structure in the solution was measured by Nanodrop (NanoDrop 2000C Spectrophotometer, Thermo Scientific) in 260 nm absorbance.

Actuation

Purified individual structures with group 1 or group 2 overhangs were mixed with 20× excess AEI and BKI conjugates relative to the structure concentrations, and then the final concentration of structures was adjusted to 5 nM with buffer containing 1× FOB and 20 mM MgCl2 and final volume is 50 µl. The mixture was subjected to an incubation of 15 hours at 37 ^{o}C.

TEM and analysis

The sample was diluted to 1 nM in 0.5× TBE with 10 mM MgCl2 buffer for transmission electron microscopy (TEM). For TEM grid preparation, 4 µl of sample volume was deposited on Formvar-coated copper TEM grids, stabilized with evaporated carbon film (Ted Pella; Redding, CA). Then the sample was incubated on the grid for 4 min when incubating single structure, and for 8 min when incubating self-assembly structures, and then was wicked away with filter paper. The sample was then stained with 2% uranyl formate (SPI, West Chester, PA, USA). First, a 10 µL drop was applied for 2 s and wicked away to wash the sample, and then another 10 µL drop was applied for 15 s and then wicked away with filter paper. TEM imaging was performed at the OSU Campus Microscopy and Imaging Facility on an FEI Tecnai G2 Spirit TEM using an acceleration voltage of 80 kV at different magnifications.

The raw TEM images were organized into a gallery containing clear formations (Figure S2-S6,S26-S28) by using the particle picking tool in software EMAN2. Then the angles were measured in the software ImageJ by drawing two straight lines directly on each particle along the inner side of each arm in one hinge.

We used MATLAB as the postprocessing tool to convert the angle data sets to probability density histograms. To estimate the fraction of the hinge in non-actuated or actuated states, we used the peakfit Matlab function program[4], which uses a non-linear optimization algorithm to decompose a complex, overlapping-peak signal into its component parts, by assuming the conformational distribution consisting of two populations. The peak number was set as 2 for the actuated sample and the distribution was fit with a combination of two Gaussian distribution, termed as the actuated and non-actuated parts.

Self-assembly

Purified individual structures with each designed overhang arrangement were mixed with 20× excess AEI and BKI conjugates relative to the structure concentrations, and then the final concentration of structures was adjusted to 5 nM with buffer containing 1× FOB and 20 mM MgCl2 and final volume is 50 µl. For the linear self-assembly polymer, the mixtures were then subjected to a 1-cycle low temperature annealing ramp starting at 45 °C followed by an anneal phase at −2 hours per °C until 20 °C, to prevent extensive aggregation. For other self-assembly structures, the process was 2-cycle annealing ramp, which was repeated twice. In Figure 2 and Figure 3 A, we used 2 cycle annealing ramp. However, for comparison of assembly with between DNA and coiled-coil peptides and polymers actuation, we used 1 cycle annealing ramp to avoid aggregation.

Circle pattern actuation

Before self-assembly process, the purified structures were mixed with 0.5 µl of 10µM closing strands and then incubated at 37 $^{\circ}$C for 15 hours to yield an angle of hinges mostly actuated to 45°.

Linear polymer actuation

After self-assembly process, 0.5 µl of 10µM closing strands were added to the production. And then the mixture was incubated to 37 $^{\circ}$C for 15 hours.

Persistence length from shape variance in TEM images

TEM images of non-actuated and actuated self-assembly polymers were analyzed using MATLAB. To discretize the shape of the polymers, 11 points on the connection of the hinge along the trajectory were manually selected by clicking on the image Figure S17A and Figure S18A These selected points were used to fit a cubic spline of the trajectory coordinates along the filament path to obtain fine resolution of the curvature. Configurational distributions were obtained by aligning the filament trajectories so that they started at the origin and initially pointed in the horizontal direction, as in Figure 3E. Isambert et al. derived a relation between the filament persistence length (LP) and the average transverse fluctuations, $\langle [D(s)]^2 \rangle$,

$$\langle [D(s)]^2 \rangle = L_p^2 \left[2\frac{s}{L_p} + \frac{16}{3}\exp\left(-\frac{s}{2L_p}\right) - \frac{1}{3}\exp\left(-\frac{2s}{L_p}\right) - 5 \right] \qquad (1)$$

The average transverse fluctuations were determined as a function of arc length from the filament configurational distributions. The LP of the actuated and non-actuated self-assembly polymer were characterized respectively by calculating the average of the transverse fluctuations squared from the configurational distributions and fitting Eq. (1).

Materials and supplies: Fmoc-protected amino acids for peptide synthesis were purchased from EDM Millipore. Fmoc-azidolysine was purchased from Combi-Blocks Inc. Dichloromethane (DCM) was purchased from Millipore Sigma. Dimethylformamide (DMF) and diethyl ether were purchased from Oakwood Chemical Inc. Piperidine was purchased from Alfa Aesar. Oxyma and DIC were purchased from ChemImpex. TFA was purchased from Oakwood, and Rink Amide resin was purchased from Novabiochem. DBCO-sulfo-NHS linker was purchased from Click Chemistry Tools. All oligonucleotides were purchased from Integrated DNA Technologies.

Peptide synthesis and characterization: Peptides were obtained using solid phase peptide synthesis (SPPS) on a CEM Liberty Blue instrument. Synthesis was performed on a solid phase Rink-Amide resin (0.78 mmol/g) at a 0.1 mmol scale, using a standard Fmoc protocol and deprotected in 20% piperidine in DMF. Amino acids, coupling agents, DIC and Oxyma, were added in a 10-fold molar excess. Crude peptides were cleaved by shaking the resin by in a solution containing trifluoroacetic acid (TFA), triisopropylsilane (TIPS), and water in a ratio of 95:2.5:2.5 for 3.5 h. The resin was washed with TFA and concentrated under nitrogen. The solution was then added to 40 mL of cold diethyl ether to precipitate the peptide. The solution was centrifuged at 4200 rpm for 10 min, the supernatant was removed, and the pellet was allowed to dry overnight. The dried pellet was dissolved in a mixture of water and acetonitrile (50:50) and 0.1% TFA. Peptides were purified via reverse phase chromatography on a Waters HPLC using a Phenomenex column with C18 resin. A linear gradient was generated using water/acetonitrile + 0.1% TFA, from 10% to 100% acetonitrile over 50 minutes. Peak fractions were collected based upon their absorbance at 230 nm and tested for purity by MALDI-TOF mass spectrometry on a Bruker Microflex LRF MALDI using α-cyano-4-hydroxycinnamic acid matrix (Sigma). Pure fractions were pooled and lyophilized, and peptides were stored at -20 °C until use.

Synthesis of Peptide-DNA Conjugates DNA-peptide conjugates were prepared via strain-promoted azide-alkyne cycloaddition (SPAAC) following reported protocols [5]. Briefly, amine modified oligonucleotides were dissolved in phosphate buffered saline (PBS) to a concentration of 1 mM. A 10 molar excess of DBCO-sulfo-NHS dissolved in DMSO was then added to the DNA and agitated at RT overnight. The reaction mixture was washed six times with a 3 kDa molecular weight cutoff (MWCO) filter (Amicon) to remove any excess DBCO. The resulting DNA was purified by reverse phase HPLC on an Agilent 1220 Infinity LC HPLC with a Zorbax Eclipse XDBC18 column. A Linear gradient was generated using 50 mM TEAA/Methanol from 10% to 100% methanol over 60 minutes. Peak fractions were collected based upon their absorbance at 260 nm and tested for purity by MALDI-TOF mass spectrometry on a Bruker Microflex LRF MALDI in 3-Hydropicolinic acid (HPA) matrix (Sigma). Pure fractions were pooled, and buffer exchanged into water using a 3 kDa MWCO filter. The purified DBCO-DNA was then mixed with the azido-peptides, heated to 37 °C, and shaken overnight. Following the reaction, samples were exchanged into water using a 3 kDa MWCO filter, and purified by reverse phase HPLC, similar to the modified DBCO-DNA.

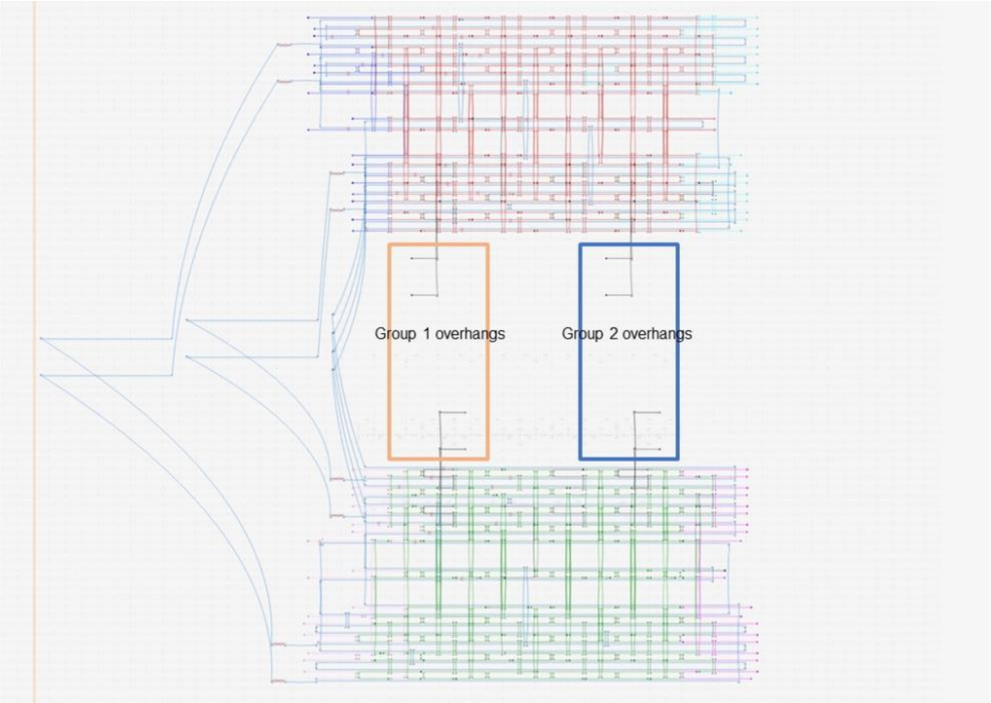

Figure S1. The caDNAno schematics of DNA origami hinge. The labeled areas are group 1 and 2 overhangs for binding AEI and BKI peptides to actuate the structure.

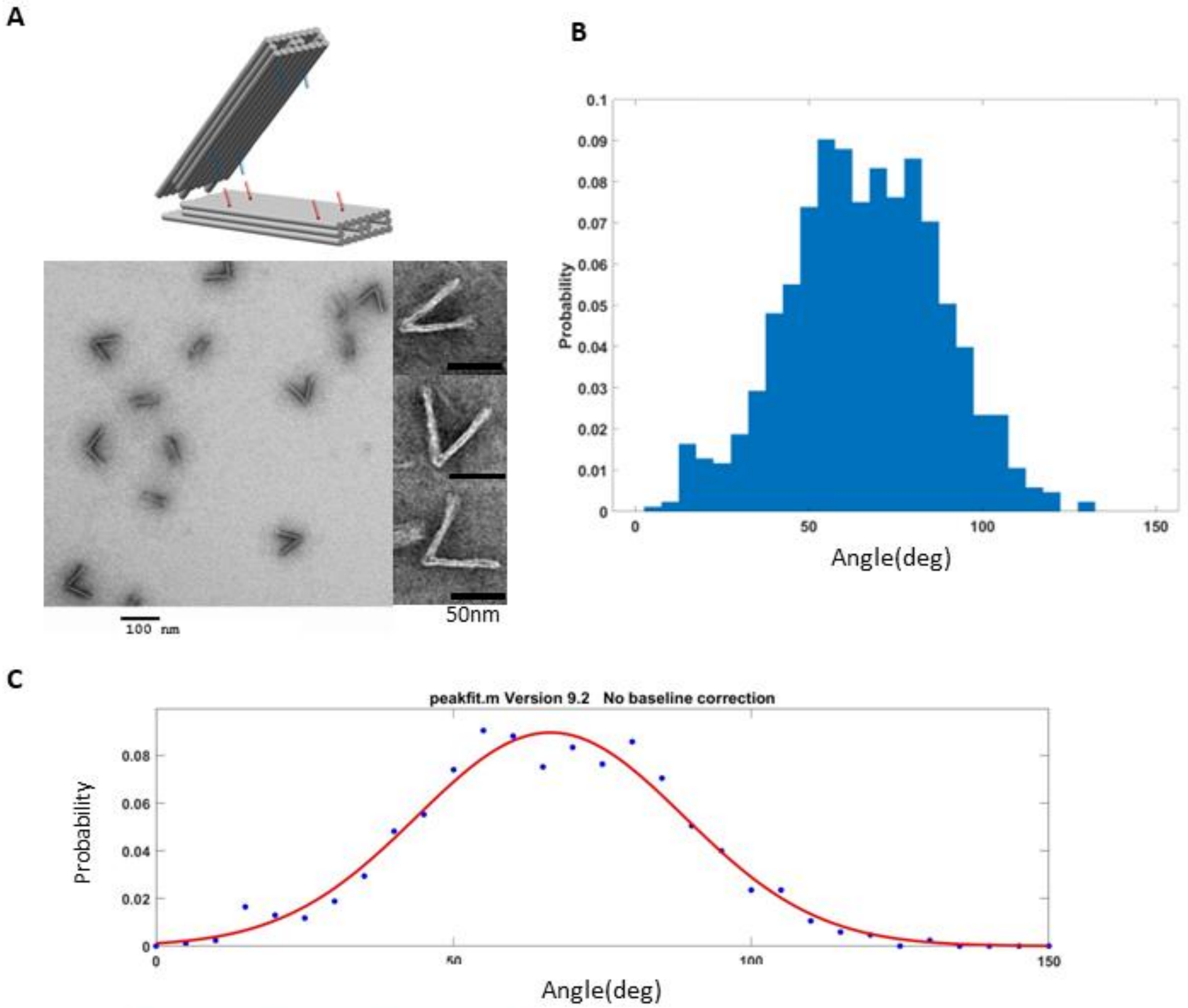

Position	Std	Area
66.2	22.4	5.0

Figure S2. Example TEM images and analysis of non-actuated DNA origami hinge. A) Example TEM images. B) The angular distributions obtained from TEM results. C) Single peak fitting of the angular distribution by using a Matlab program. The sample size is 852.

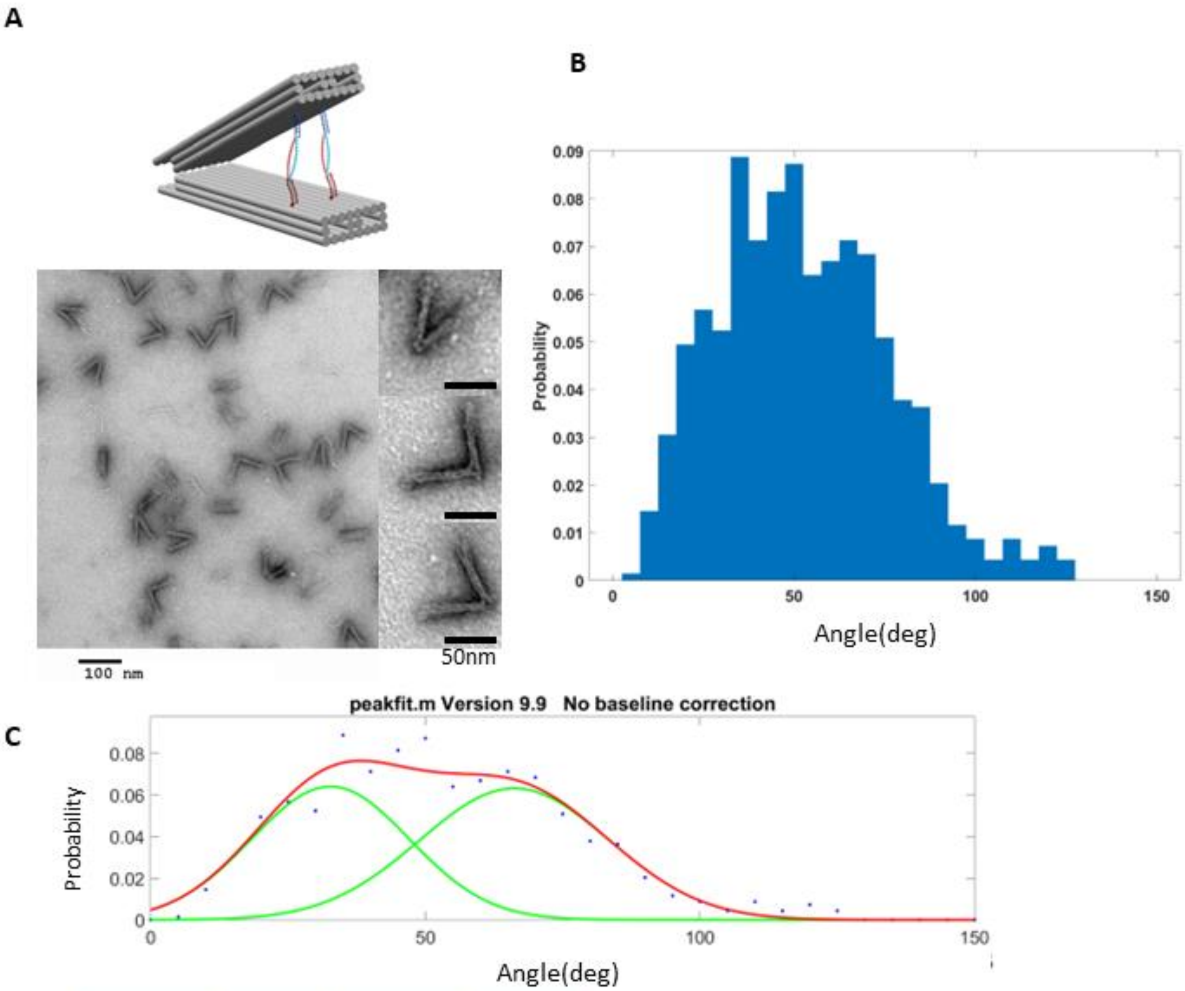

	Position	Std	Area
High peak	66.2	17.2	2.7
Low peak	32.7	14.4	2.3

Figure S3. Example TEM images and analysis of actuated DNA origami hinge when N-term coiled-coil peptides binding group 2 overhangs. A) Examples of TEM figures. B) The angular distributions obtained from TEM results. C) Double peaks fitting of the angular distribution. The lower peak is 32.7^{o} and the higher peak is 62.7^{o}. The sample size is 687.

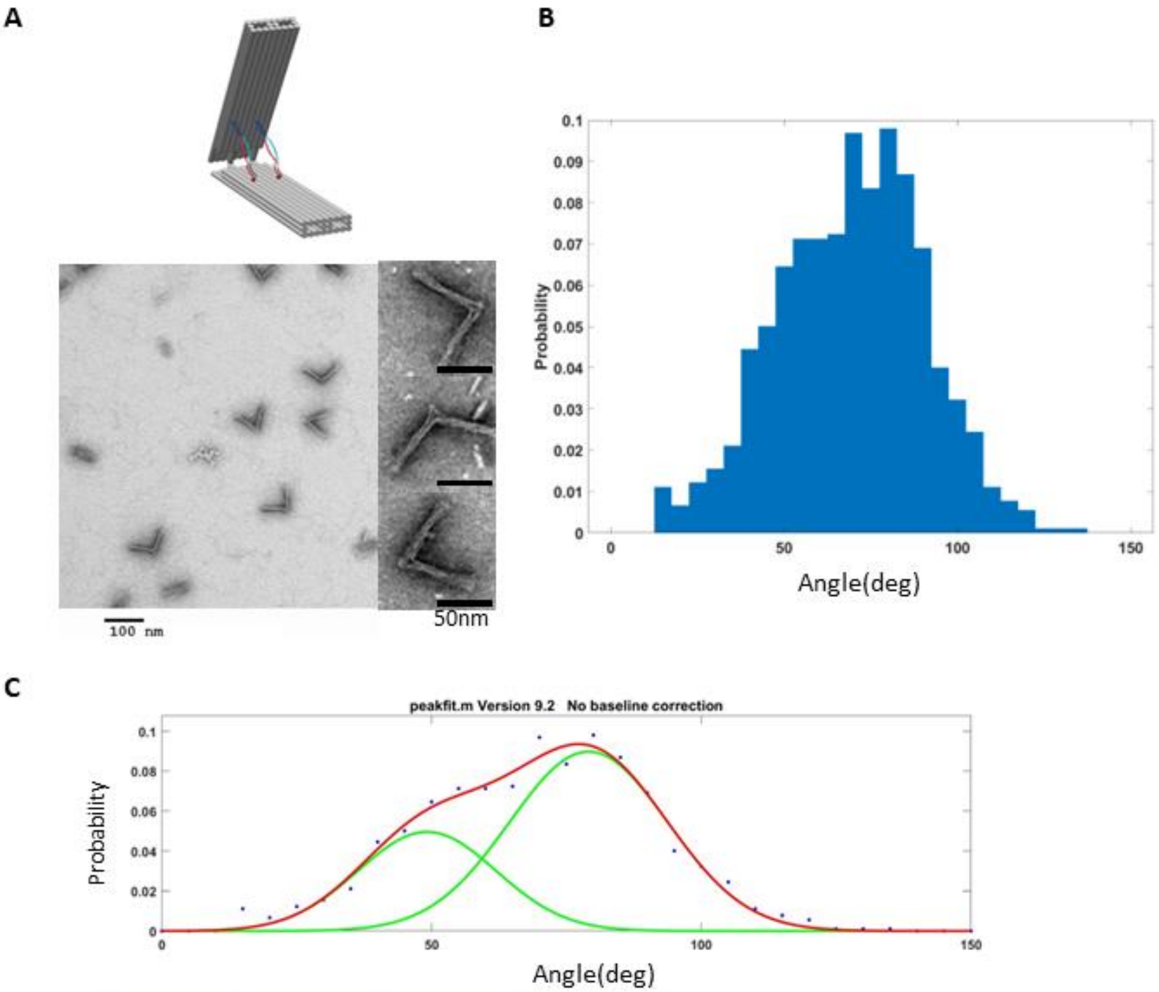

	Position	Std	Area
High peak	79.1	14.6	3.3
Low peak	49.1	12.8	1.5

Figure S4. Example TEM images and analysis of actuated DNA origami hinge when N-term coiled-coil peptides binding group 1 overhangs. A) Example TEM images. B) The angular distributions obtained from TEM results. C) Double peaks fit of the angular distribution. The lower peak is 49.2^{o} and the higher peak is 79.1^{o}. The sample size is 898.

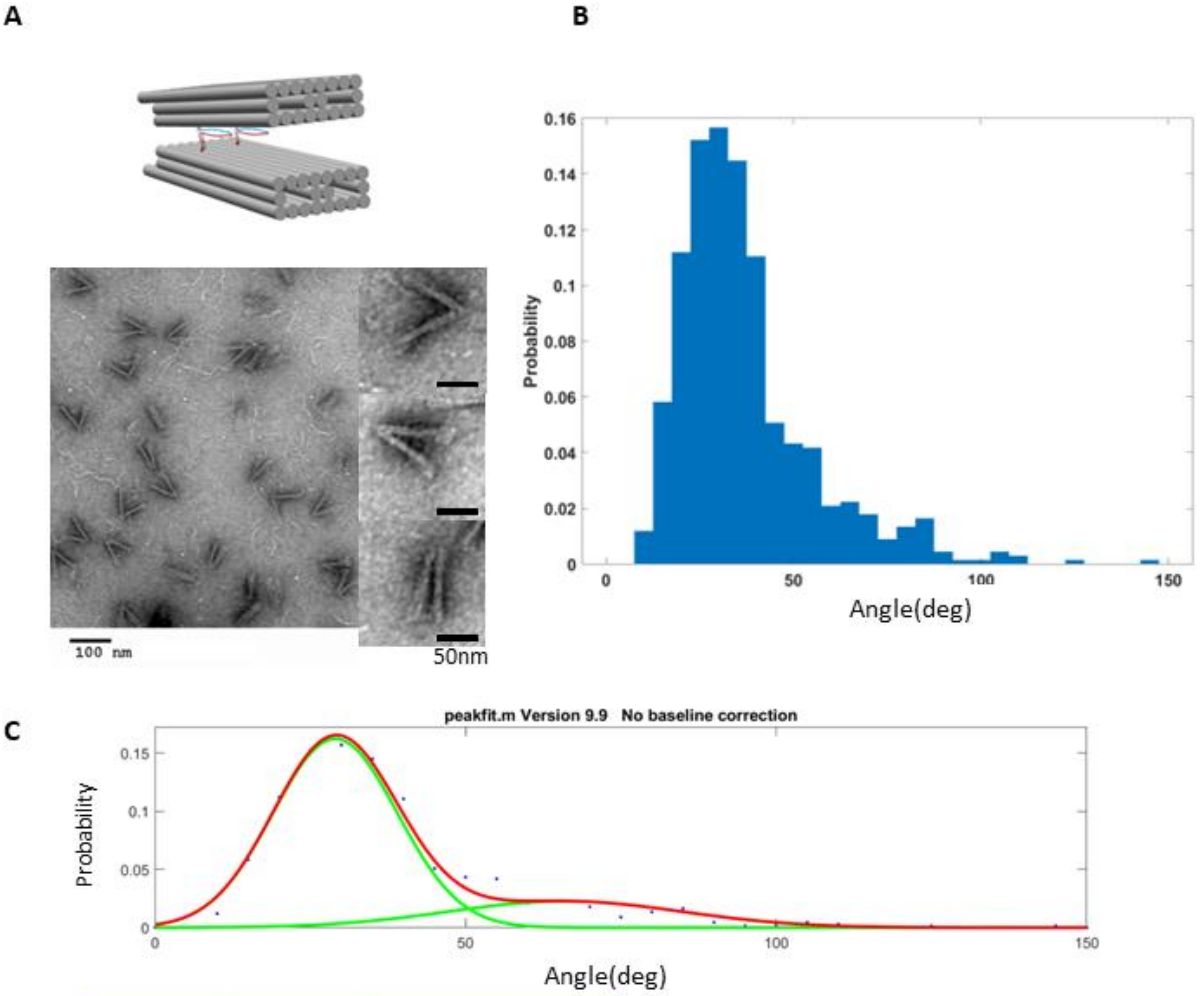

	Position	Std	Area
High peak	66	18.8	1.3
Low peak	29	10.0	3.6

Figure S5. Example TEM images and analysis of actuated DNA origami hinge when C-term coiled-coil peptides binding group 1 overhangs. A) Example TEM images. B) The angular distributions obtained from TEM results. C) Double peaks fitting of the angular distribution. The lower peak is 29.0^{o} and the higher peak is 54.11^{o}. The sample size is 670.

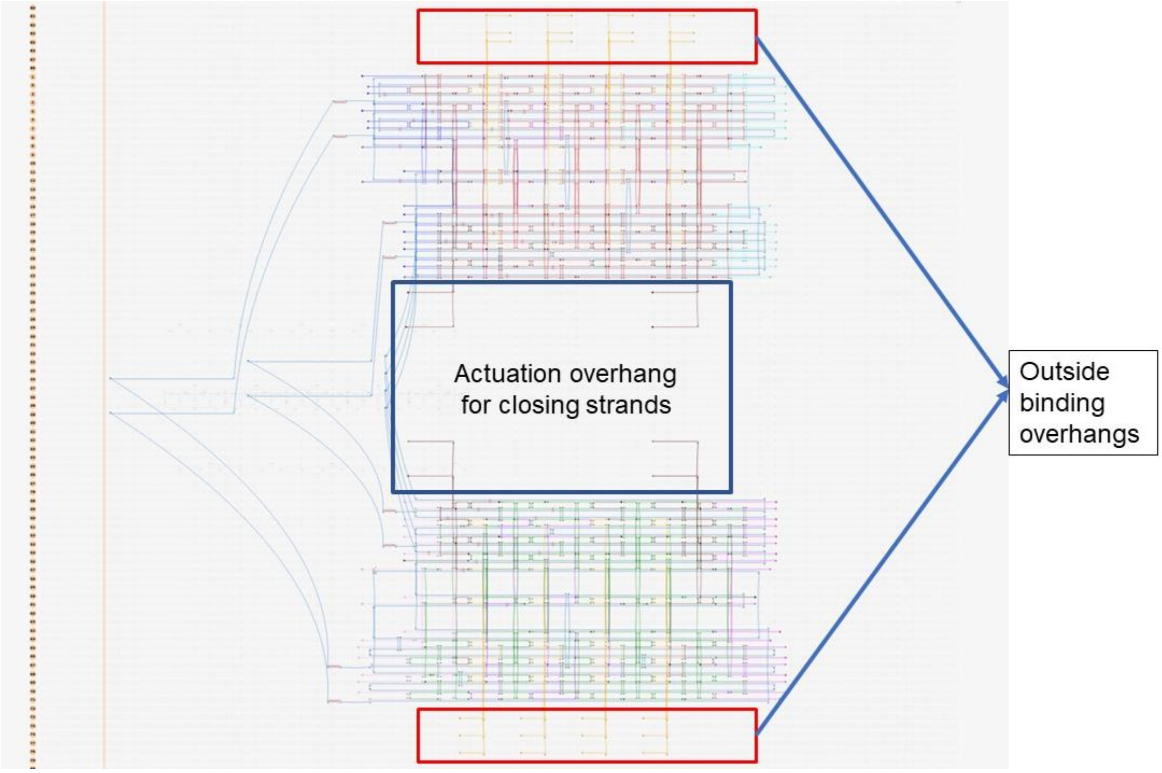

Figure S6. The caDNAno schematics of DNA origami hinge for self-assembling.

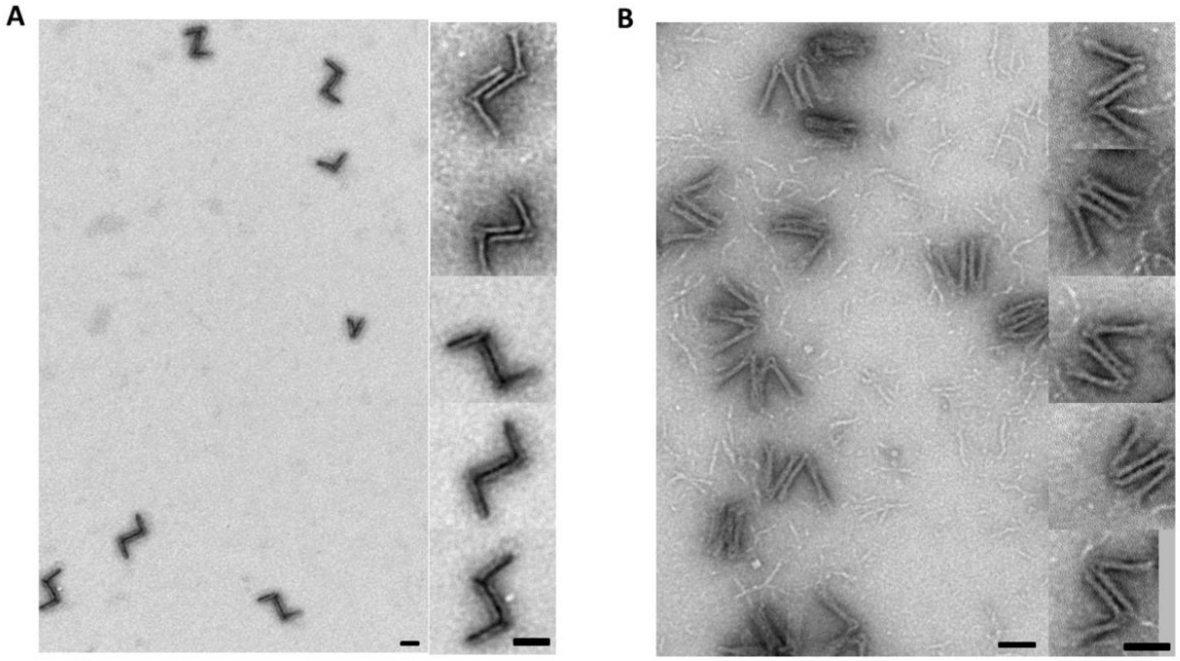

Figure S7. Example TEM images for dimers of DNA origami hinge linked by coiled-coil peptides. A) The overhangs are set on the inner side of the arm. B) The overhangs are set on the outer side of the arm. There are two different assemble directions when two hinge binding together. All scale bars are 50nm.

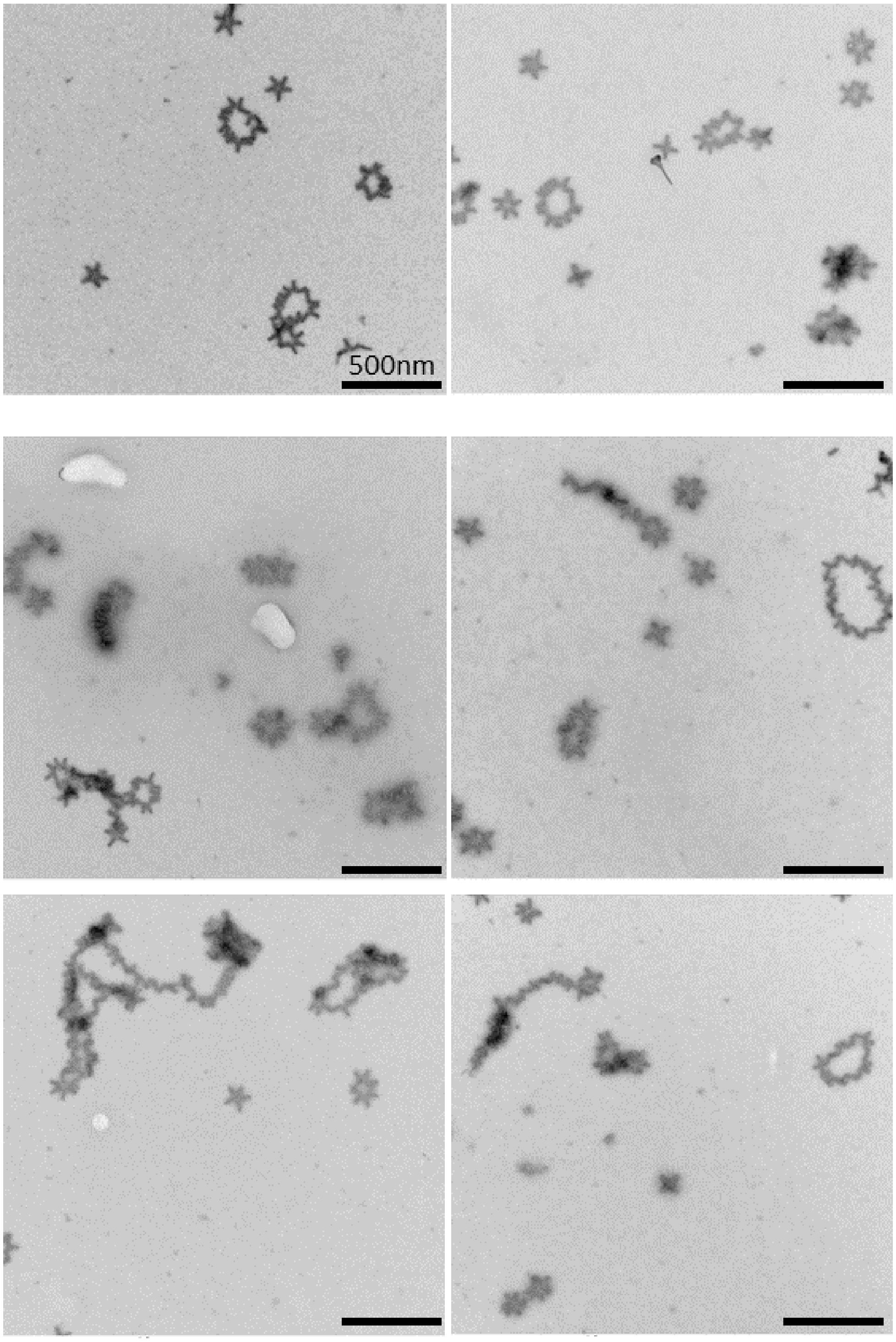

Figure S8. Representative TEM images of unrestricted self-assembly with coiled-coil peptides. Both polymers and circle patterns can be observed. All scale bars are 500nm.

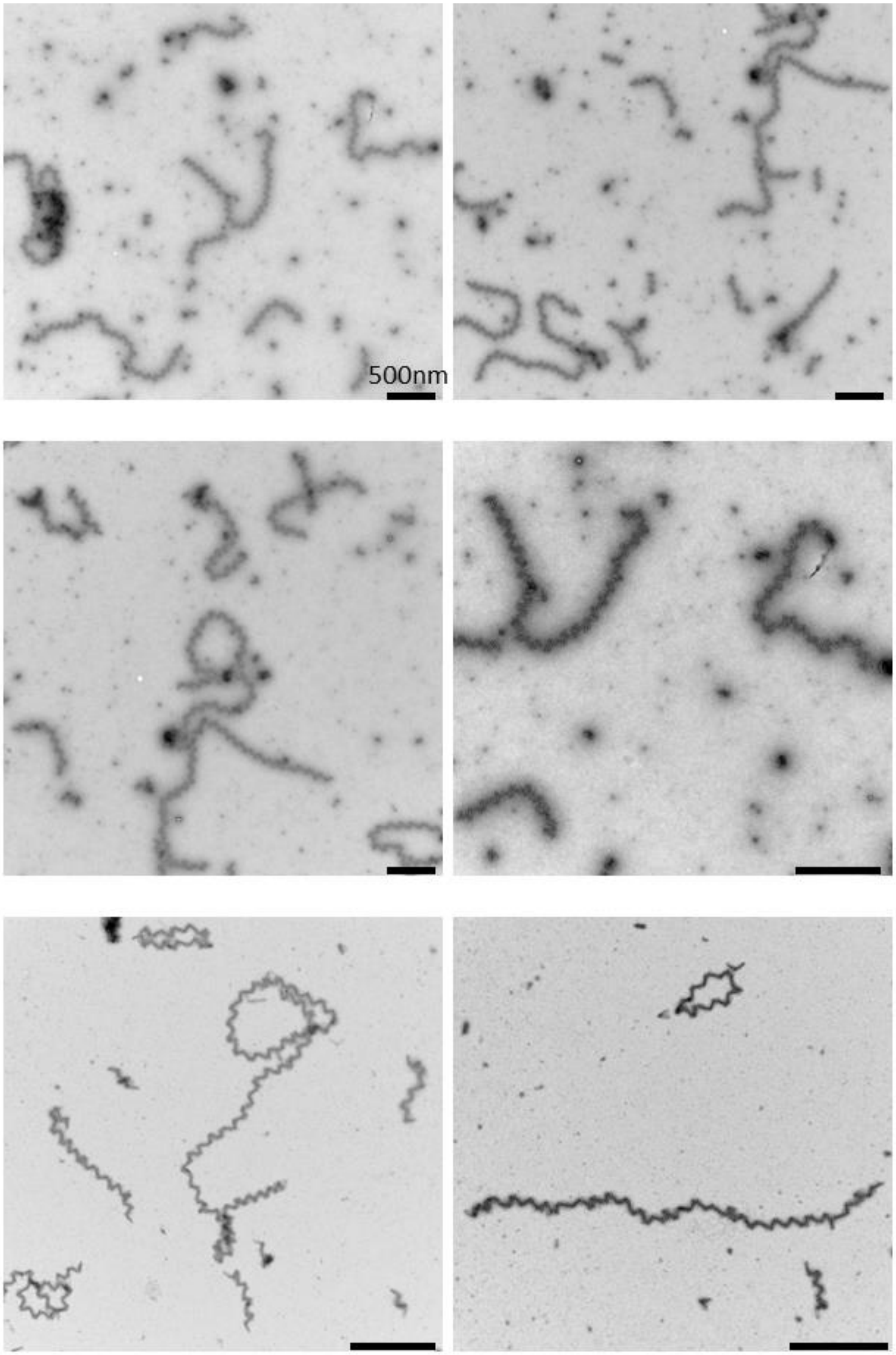

Figure S9. Representative TEM images of linear polymers self-assembly with coiled-coil peptides. All scale bars are 500nm.

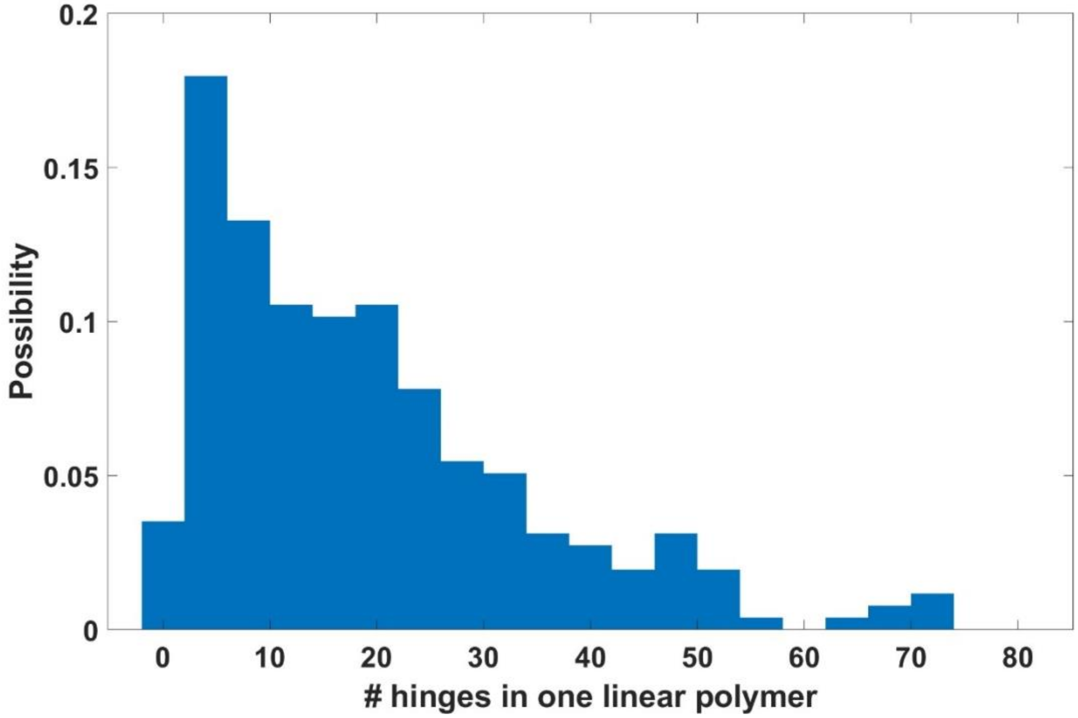

Figure S10. Length analysis of linear polymers in TEM images. This polymerization was carried out with hinges at 5 nM concentration.

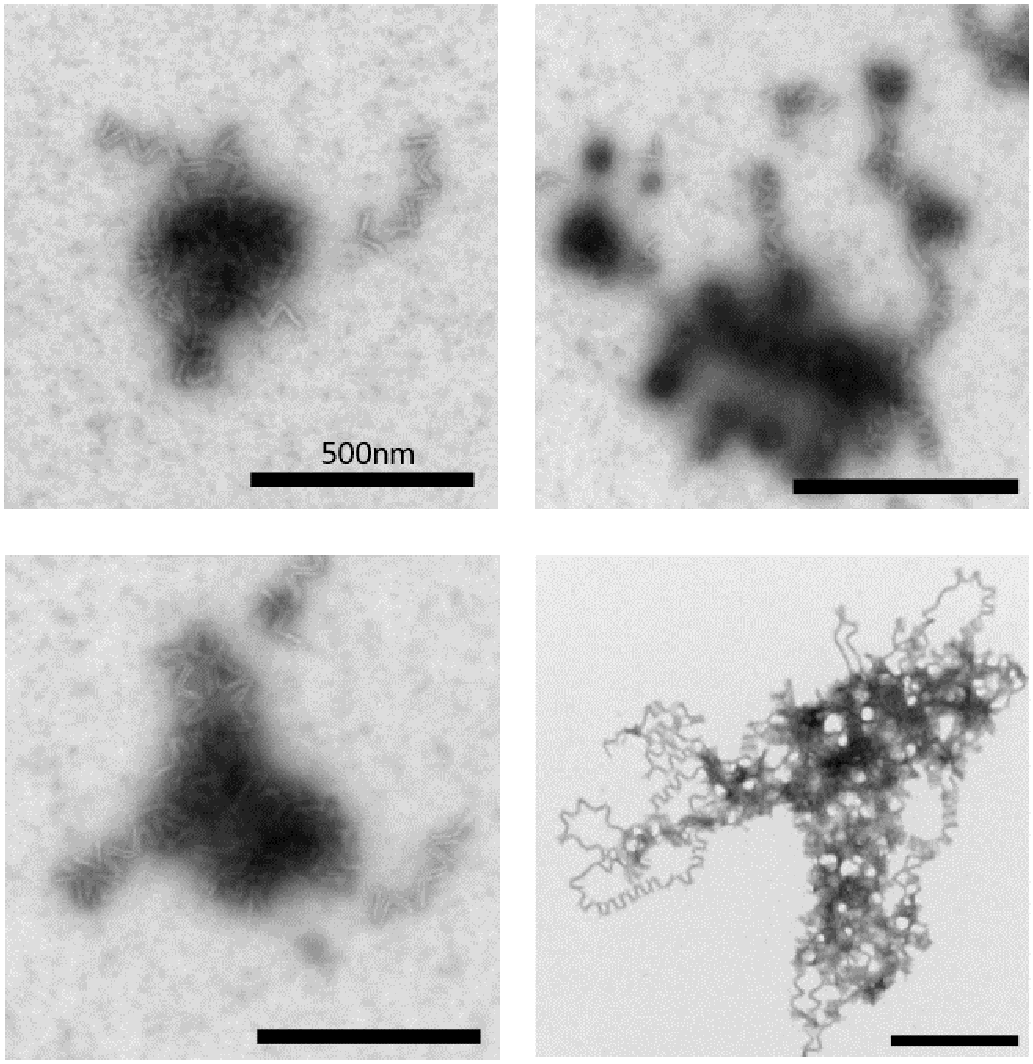

Figure S11. Representative TEM images of linear polymers self-assembly with replacement DNA strands. In linear polymer self-assembly with coiled-coil peptides, 20x access AEI and BKI were added to 5nM origami structure, and in the comparison experiment only with replacement DNA strands, 20x access of single strand DNA was added to 5nM structure. All scale bars are 500nm.

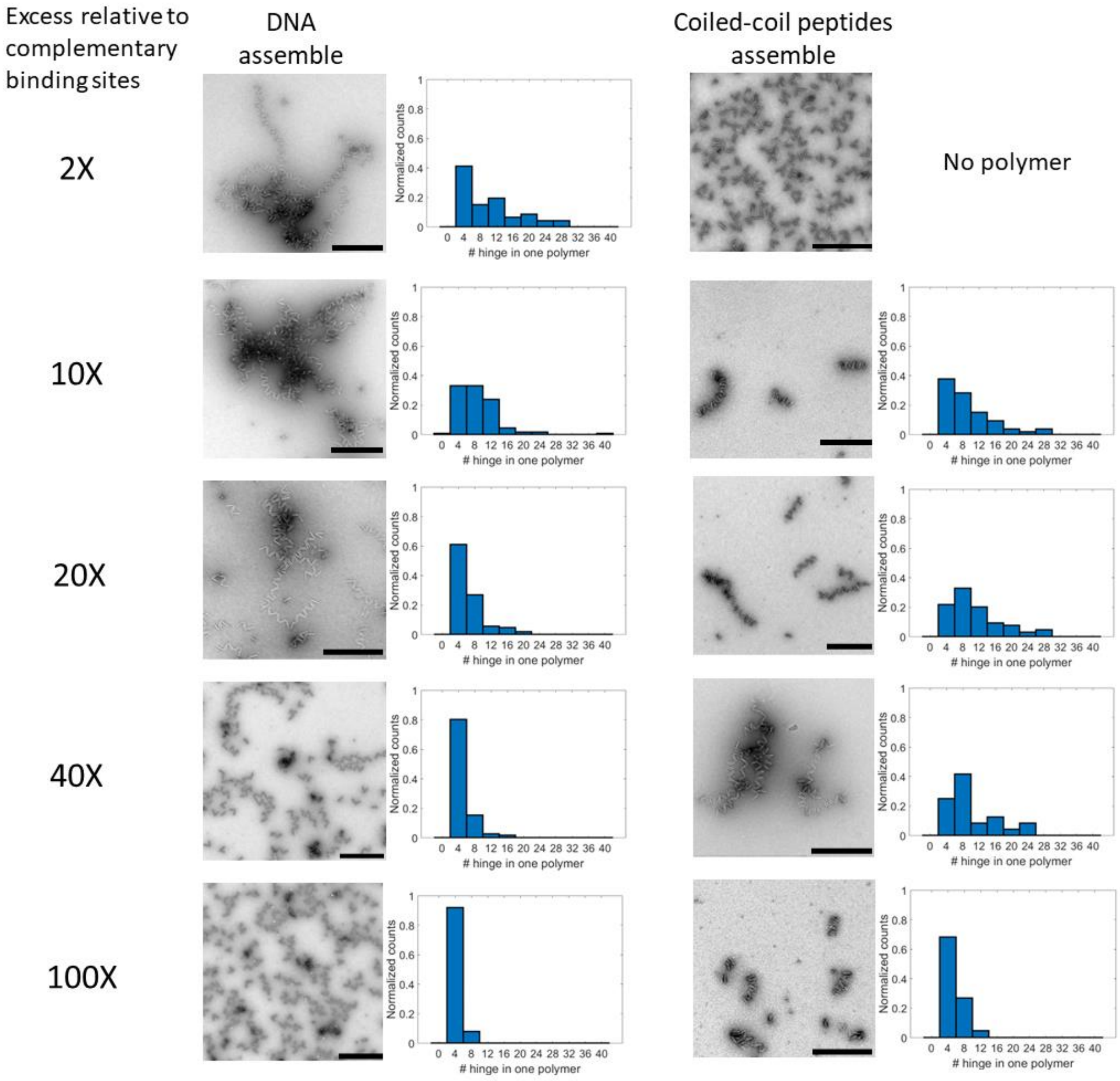

Figure S12. Experiments for assembly with different access of either DNA binding strands and peptide conjugates. In experiments, the DNA origami hinge concentration was kept at 1nM and for each experiment, DNA and peptides concentrations (excess relative to the complementary binding sites is denoted in parenthesis, there are 12 overhangs for each peptide) were set as 24nM (2X), 120nM (10X), 240nM (20X), 480nM (40X) and 1200nM (100X). The results show comparing with coiled-coil peptide conjugates, DNA can cause more aggregation even under same polymerization strand conditions. Length distributions were measured for isolated hinge assemblies (i.e. not part of a larger aggregate). These results showed that at peptide interactions yield longer unaggregated polymers, and at higher concentrations both excess DNA and coiled-coil peptides block the assembly binding sites in structures leading to a decrease in the length of polymers. This blocking occurs sooner for DNA sticky ends compared to the peptides. All scale bars are 500nm. Additional TEM images for all conditions are shown in Supplemental Figures S13-S22.

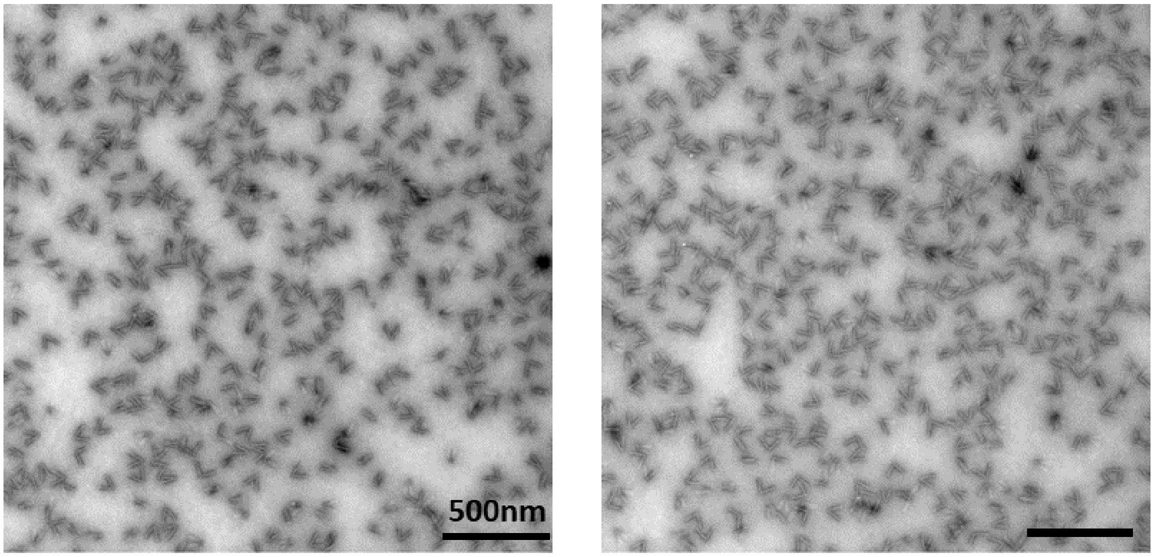

Figure S13. Example TEM images for assembly with coiled-coil peptides with relative excess of 2X. All scale bars are 500nm.

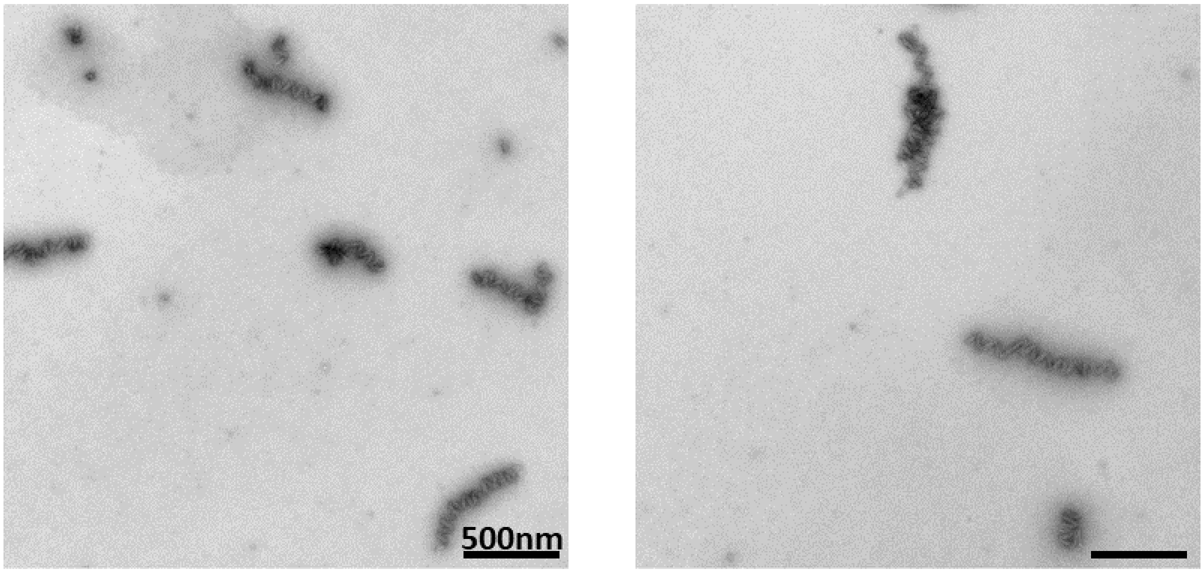

Figure S14. Example TEM images for assembly with coiled-coil peptides with relative excess of 10X. All scale bars are 500nm.

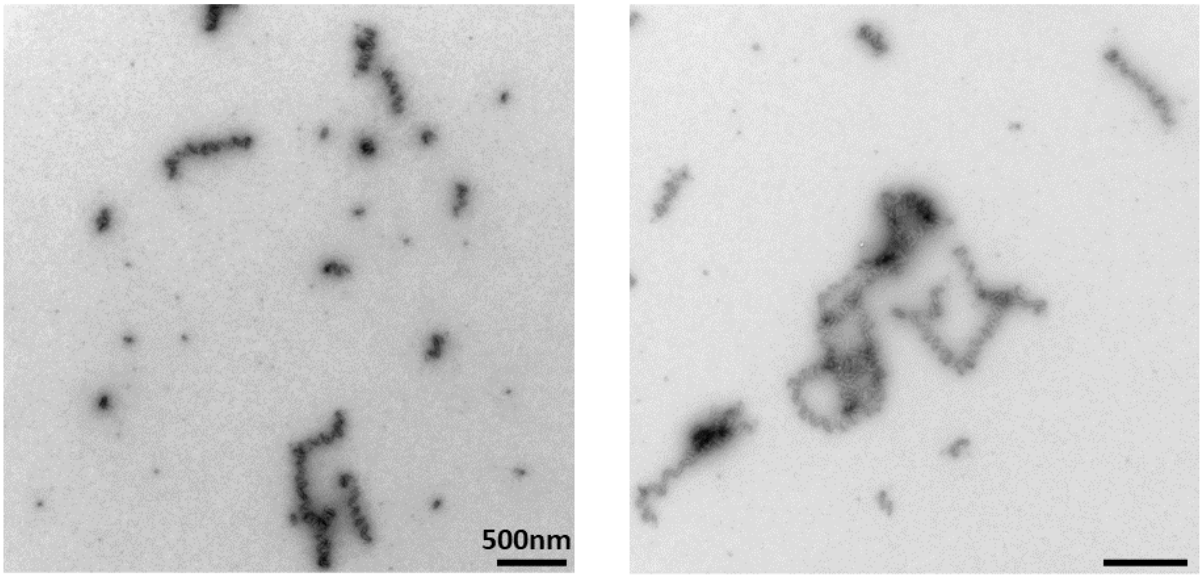

Figure S15. Example TEM images for assembly with coiled-coil peptides with relative excess 20X. All scale bars are 500nm.

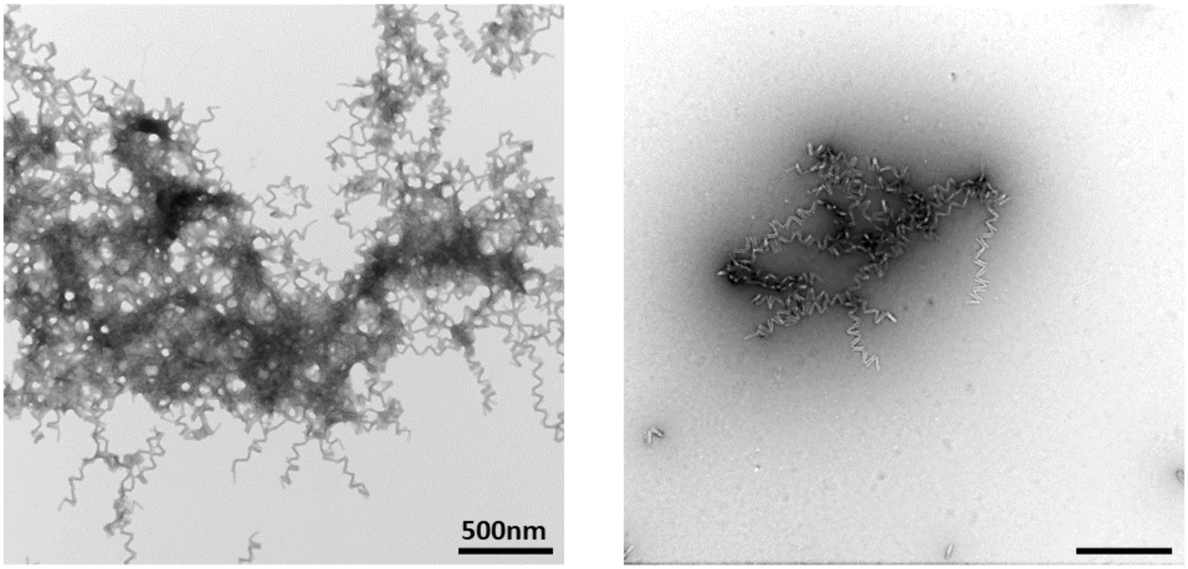

Figure S16. Example TEM images for assembly with coiled-coil peptides with relative excess 40X. All scale bars are 500nm.

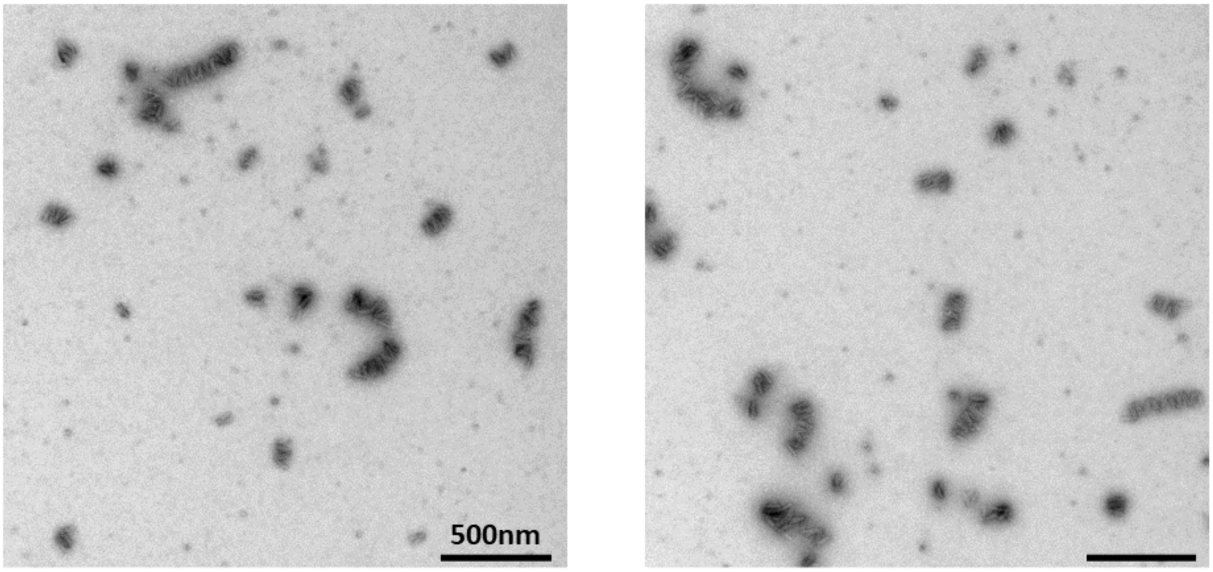

Figure S17. Example TEM images for assembly with coiled-coil peptides with relative excess 100X. All scale bars are 500nm.

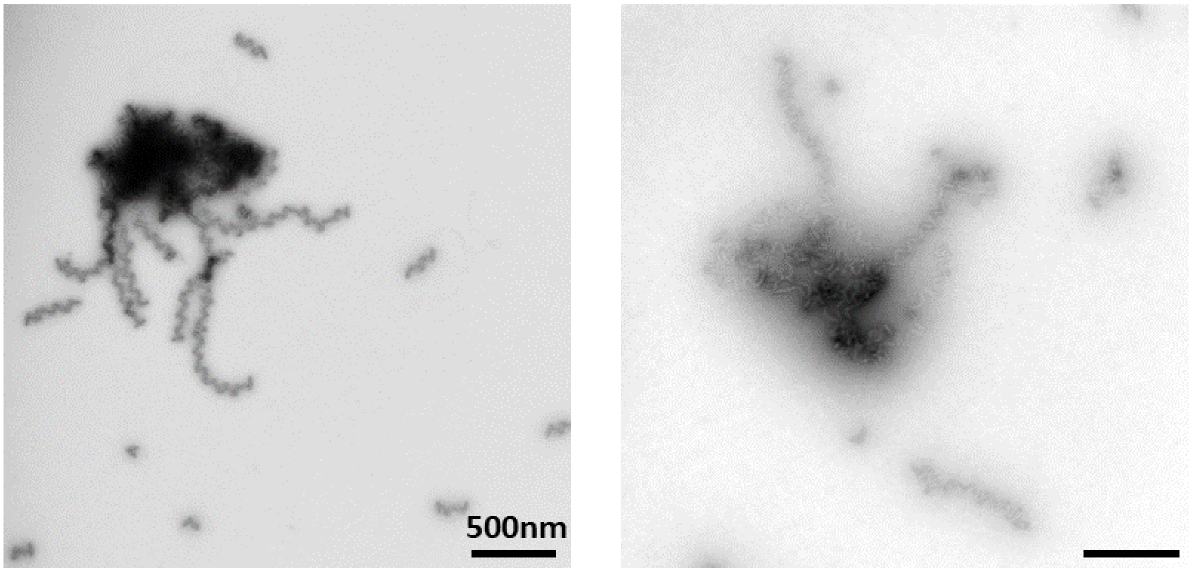

Figure S18. Example TEM images for assembly with DNA binding strand with relative excess 2X. All scale bars are 500nm.

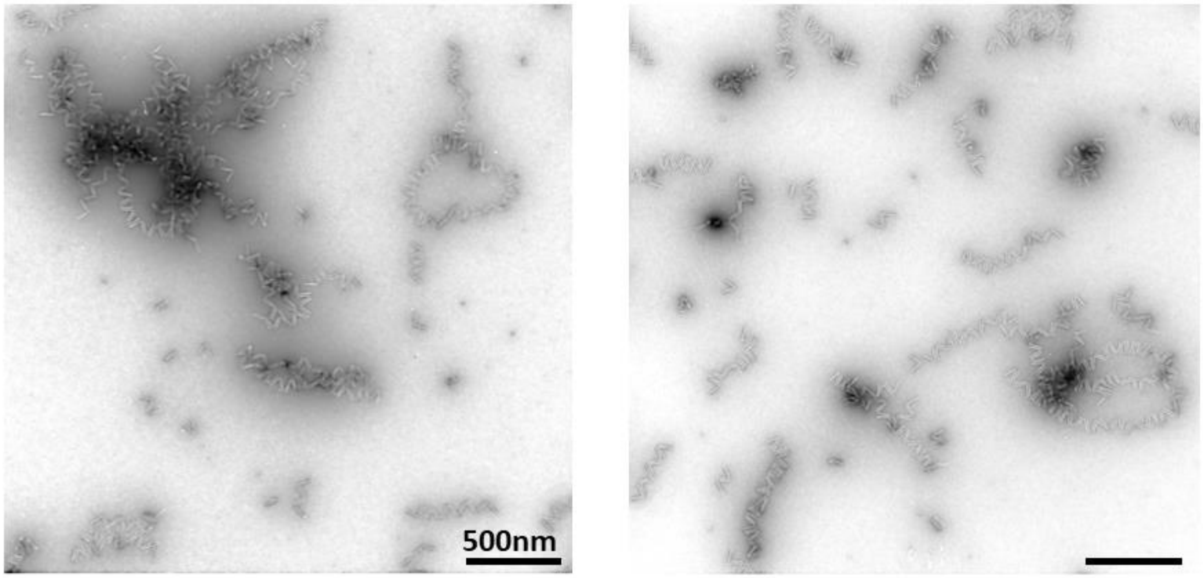

Figure S19. Example TEM images for assembly with DNA binding strand with relative excess 10X. All scale bars are 500nm.

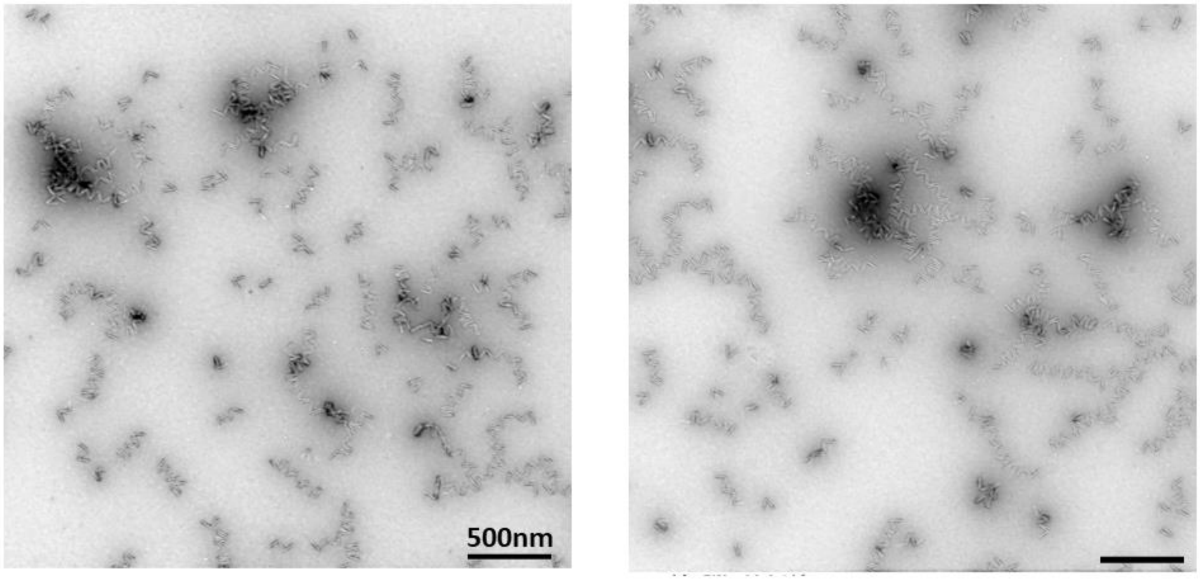

Figure S20. Example TEM images for assembly with DNA binding strand with relative excess 20X. All scale bars are 500nm.

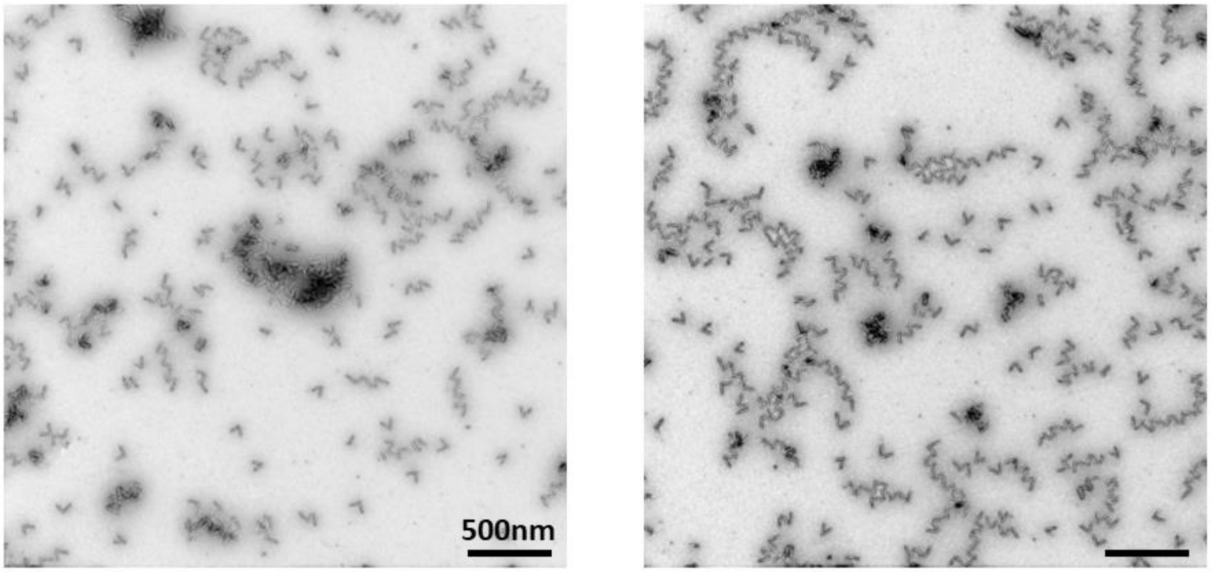

Figure S21. Example TEM images for assembly with DNA binding strand with relative excess 40X. All scale bars are 500nm.

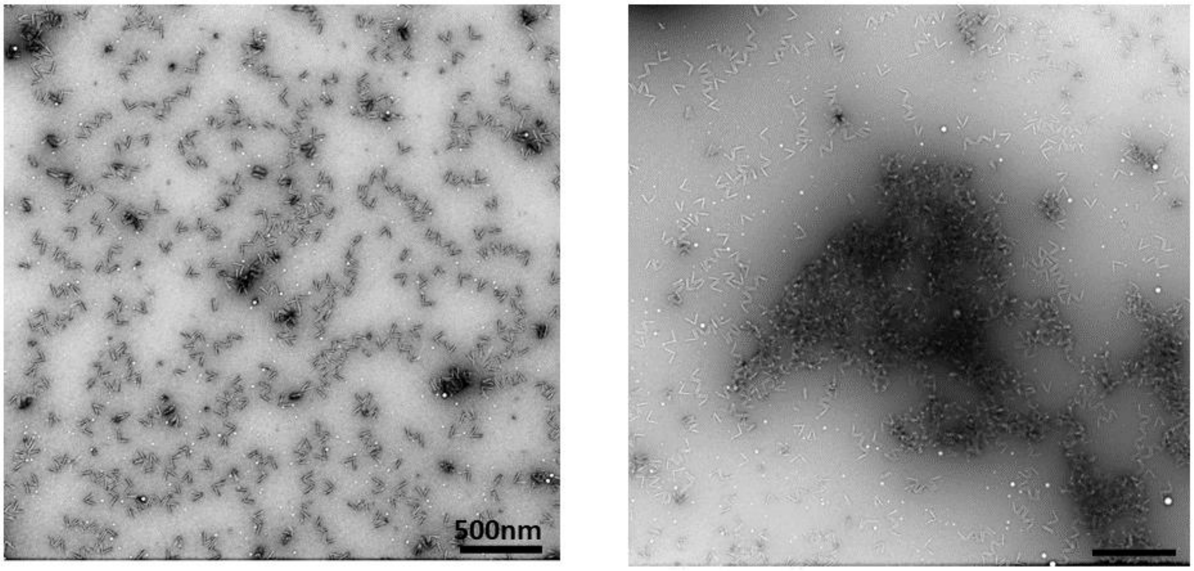

Figure S22. Example TEM images for assembly with DNA binding strand with relative excess 100X. All scale bars are 500nm.

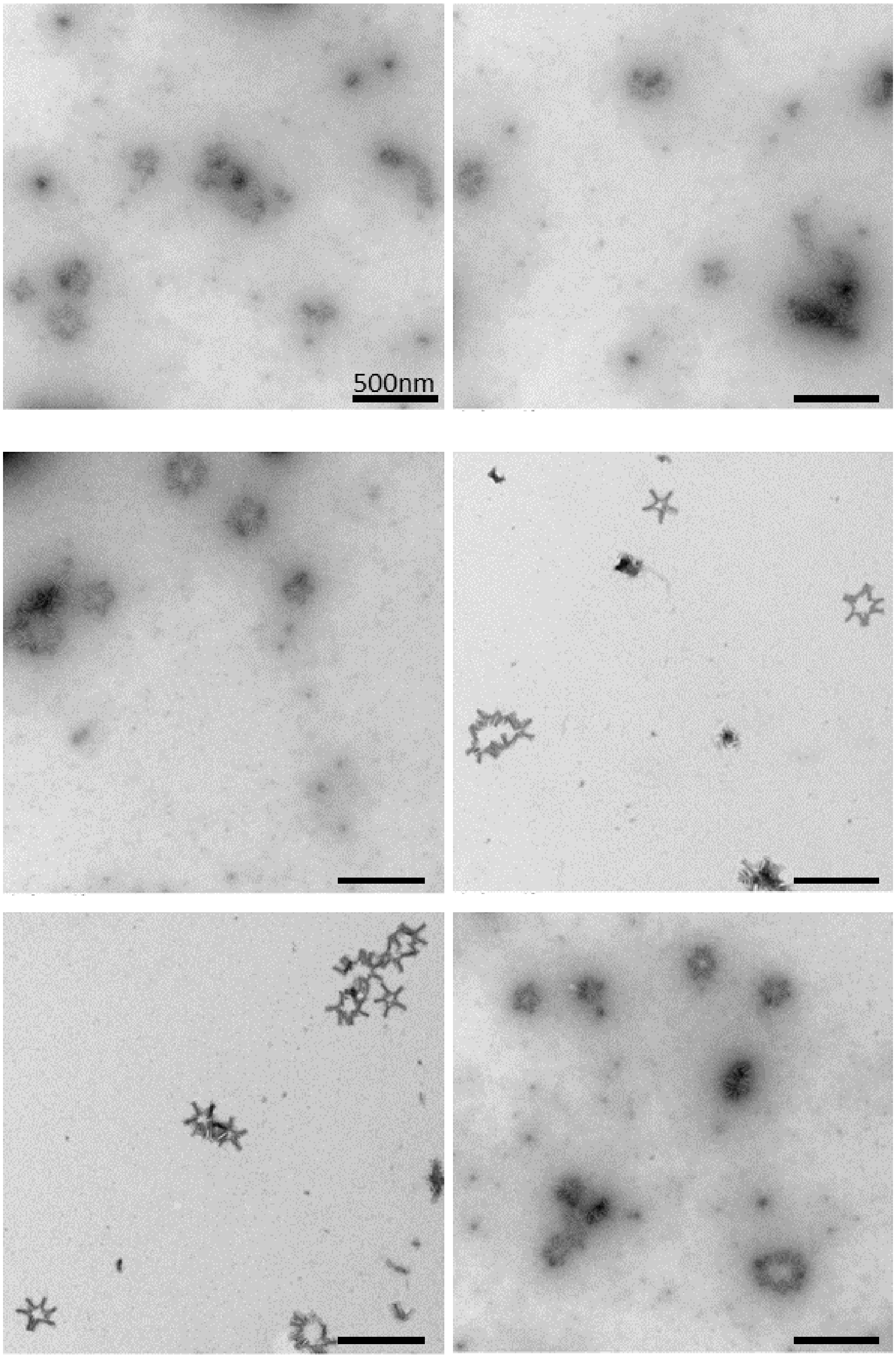

Figure S23. Representative TEM images of failed circle pattern self-assembly with coiled-coil peptides. It does not form clear circle pattern as design and both same and opposite direction assemblies happen. All scale bars are 500nm.

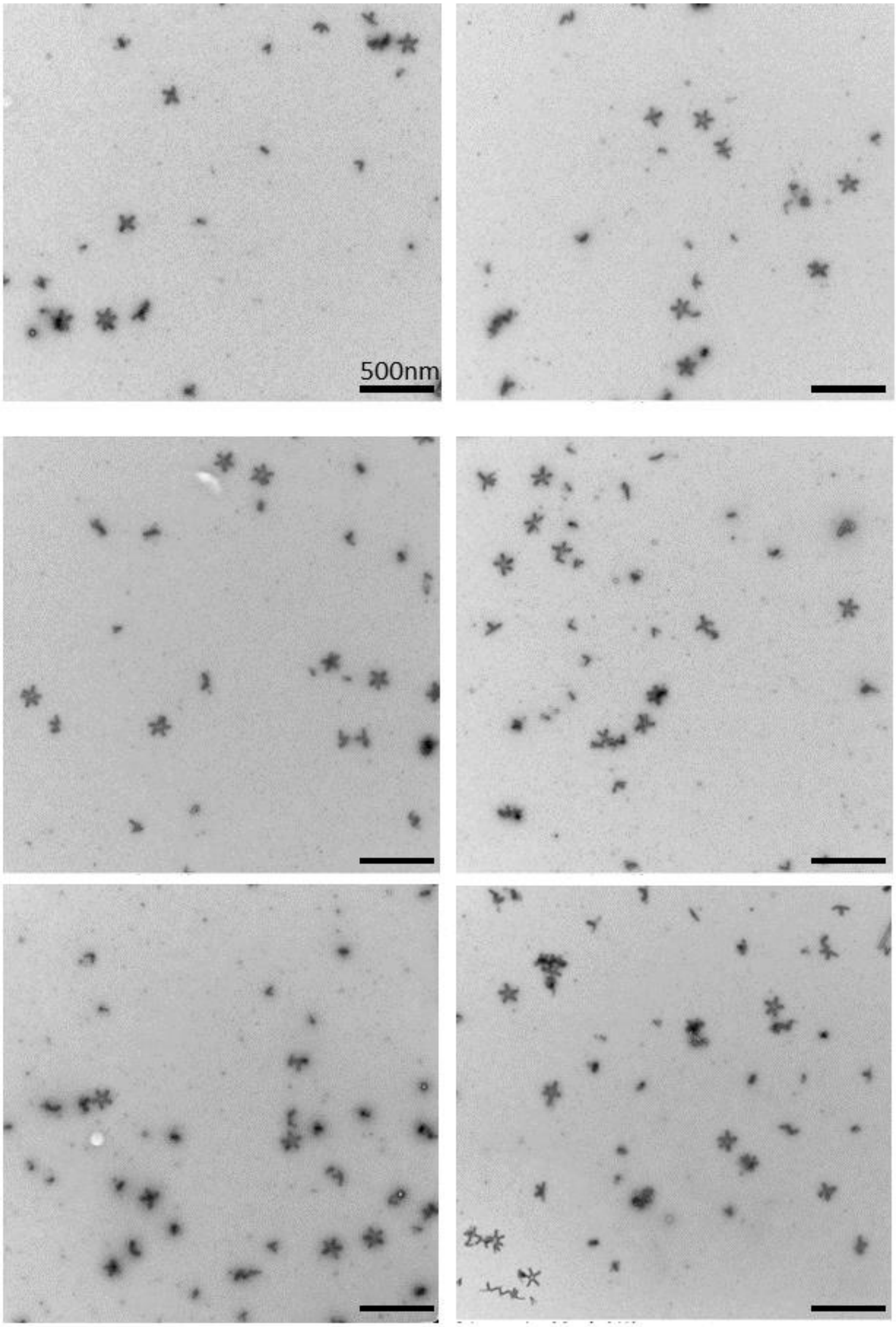

Figure S24. Representative TEM images of complexed overhangs design of circle pattern self-assembly with coiled-coil peptides. Clear circle patterns can be observed. All scale bars are 500nm.

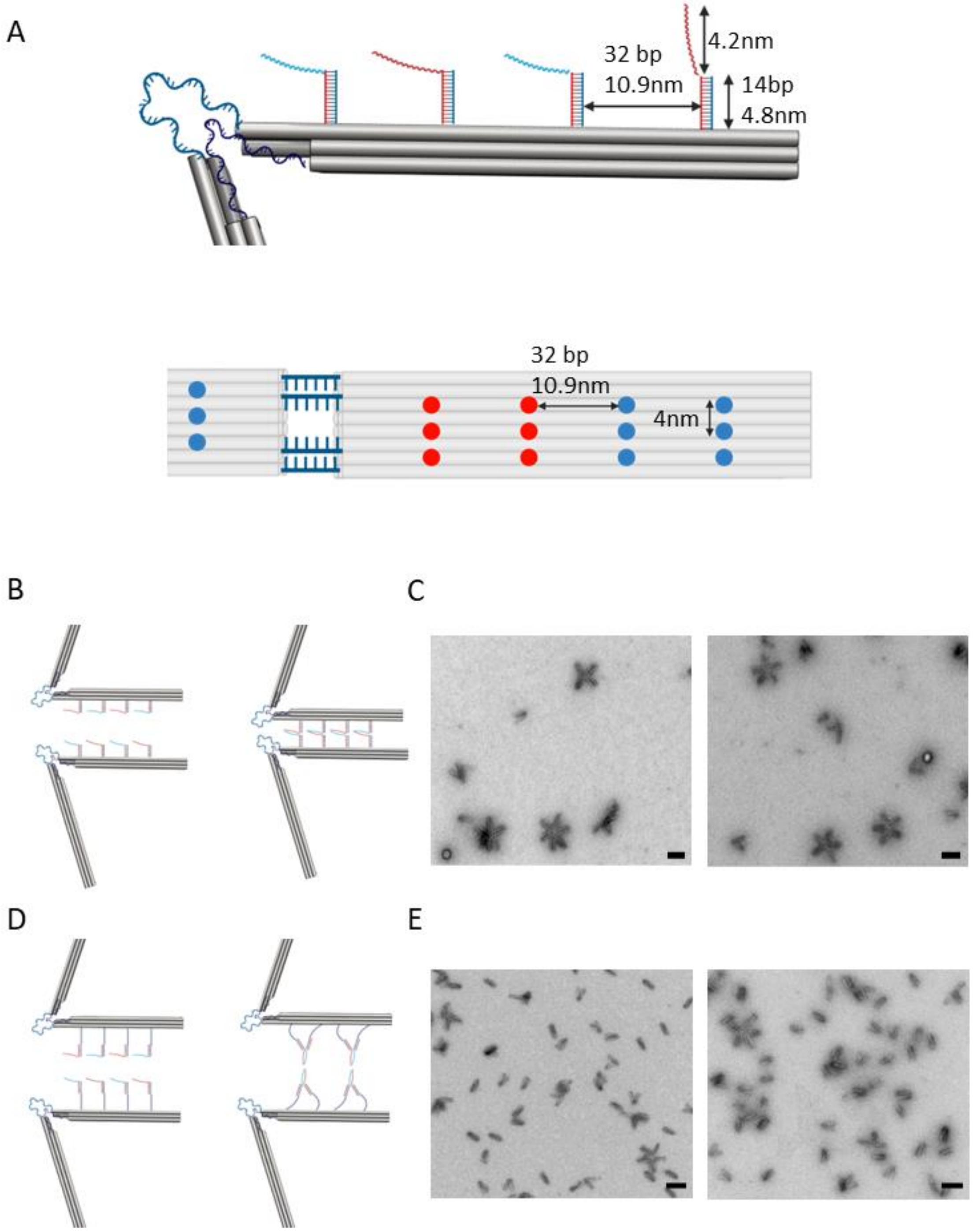

Figure S25. A) Schematics of distances between binding sites and peptides. B, C) Schematics and TEM images for normal circle pattern assembly. D, E) When the binding sites are all extended with 15 nucleotides polyT, the efficiency of assembly becomes lower. We demonstrate that it is caused by flexibility of binding sites make neighbor coiled-coil peptides binding each other. Scale bars are 100nm.

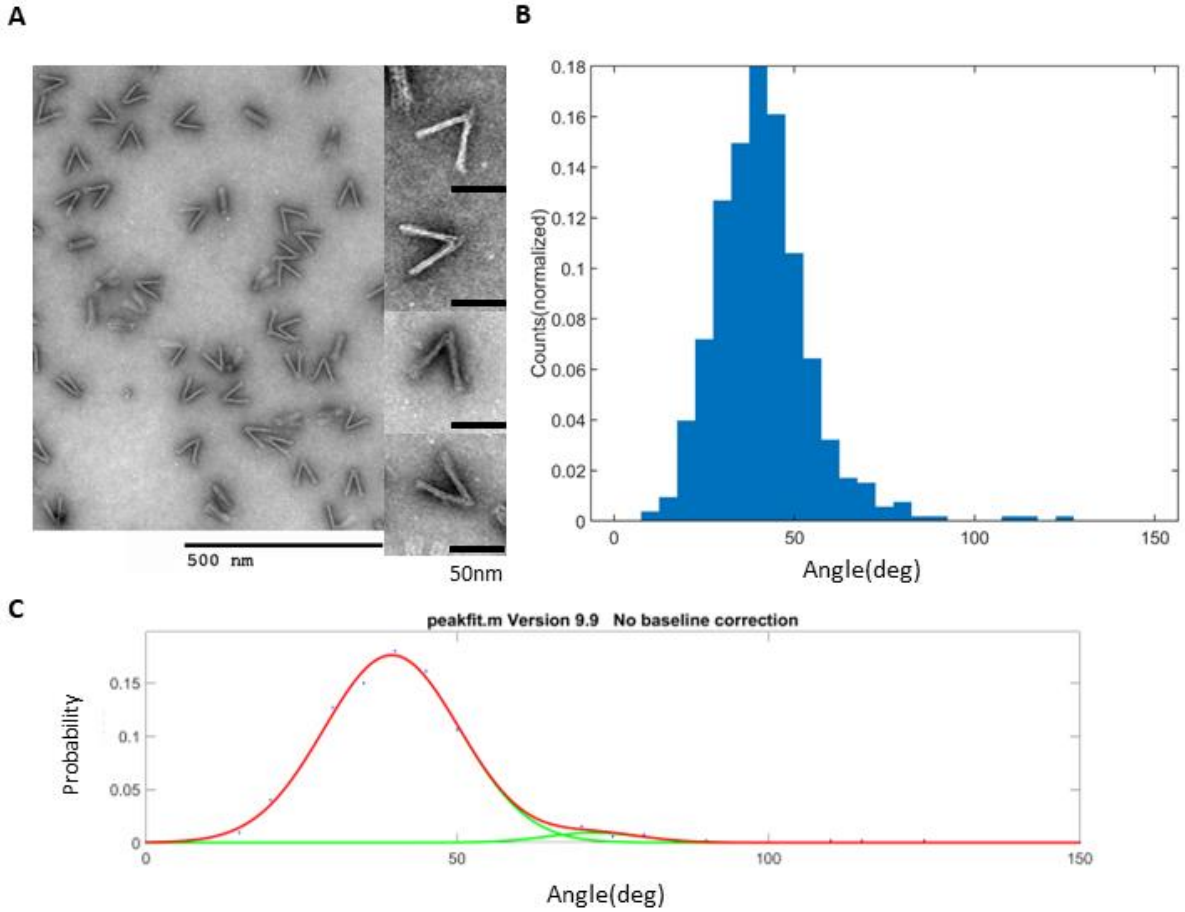

	Position
High peak	71.9
Low peak	39.5

Figure S26. Example TEM images of actuated hinge with average angle 41° before self-assembly. B) The angular distributions obtained from TEM results. C) Double peaks fit the angular distribution. The lower peak is 45°.

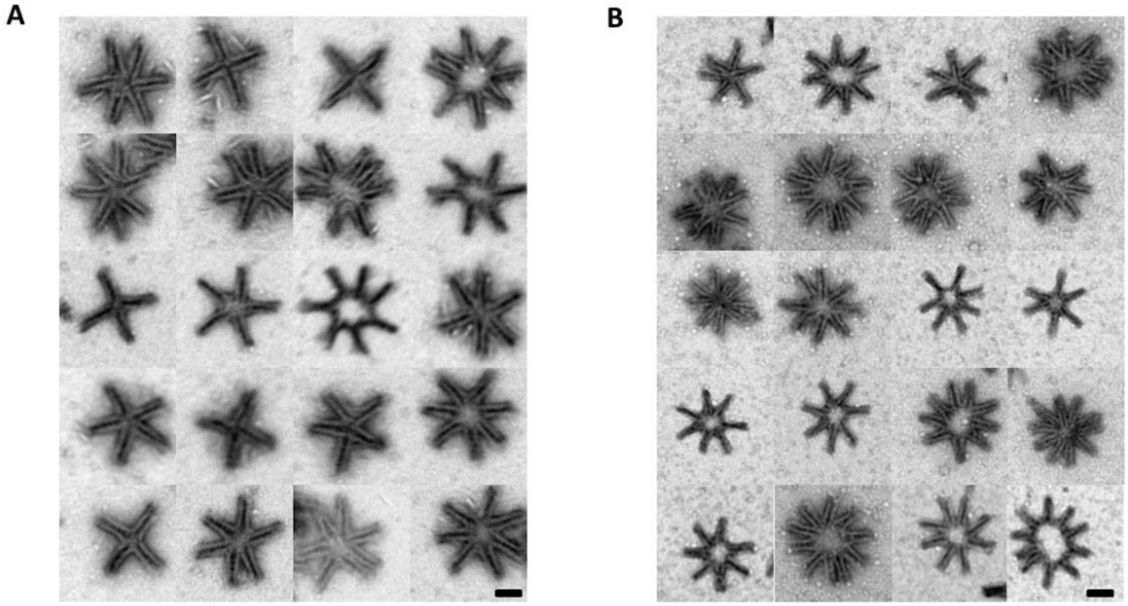

Figure S27. Example TEM images showing self-assembly of the circular pattern with A) free hinges and B) hinges reconfigured to 45°. All scale bars are 50nm.

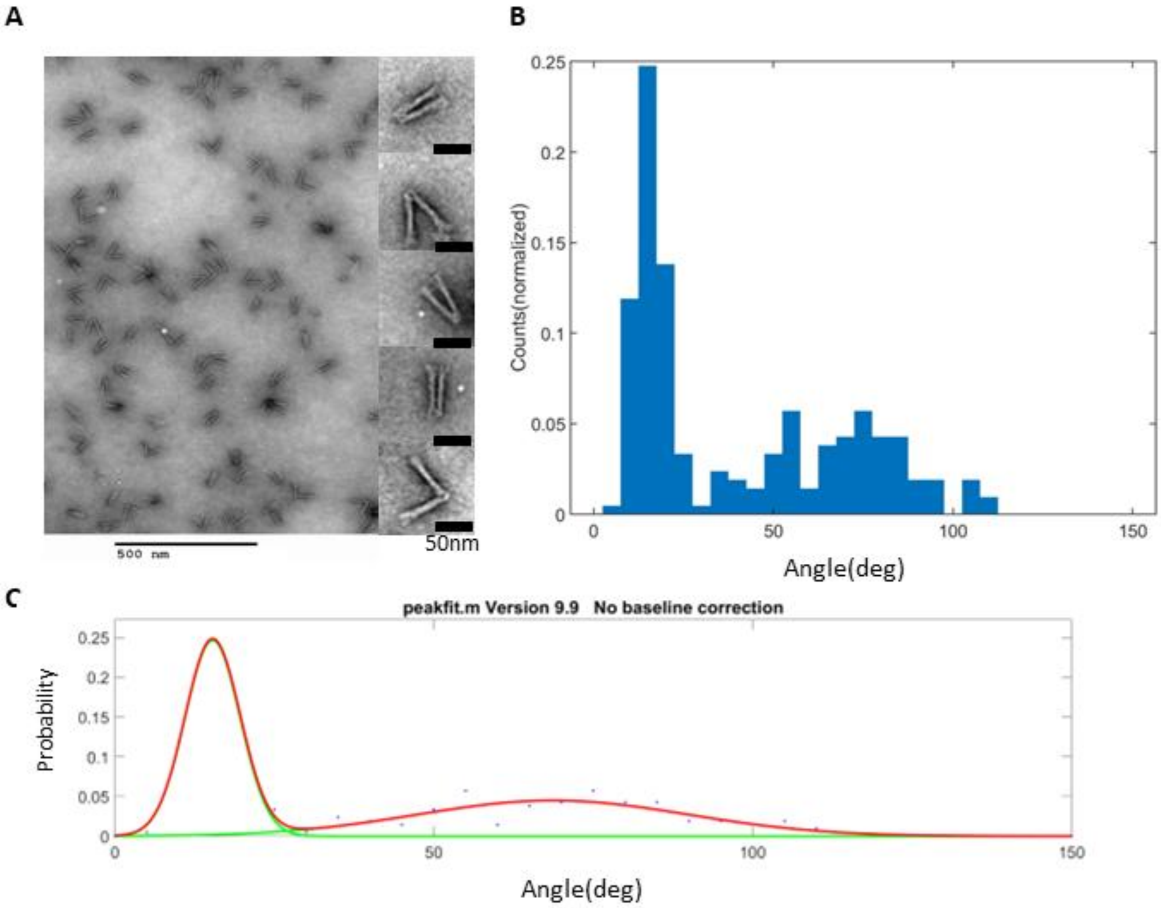

	Position
High peak	68.67
Low peak	15.32

Figure S28. Example TEM images of actuated hinge which is used in self-assemble linear polymer. B) The angular distributions obtained from TEM results. C) Double peaks fit the angular distribution. The lower peak is 15°.

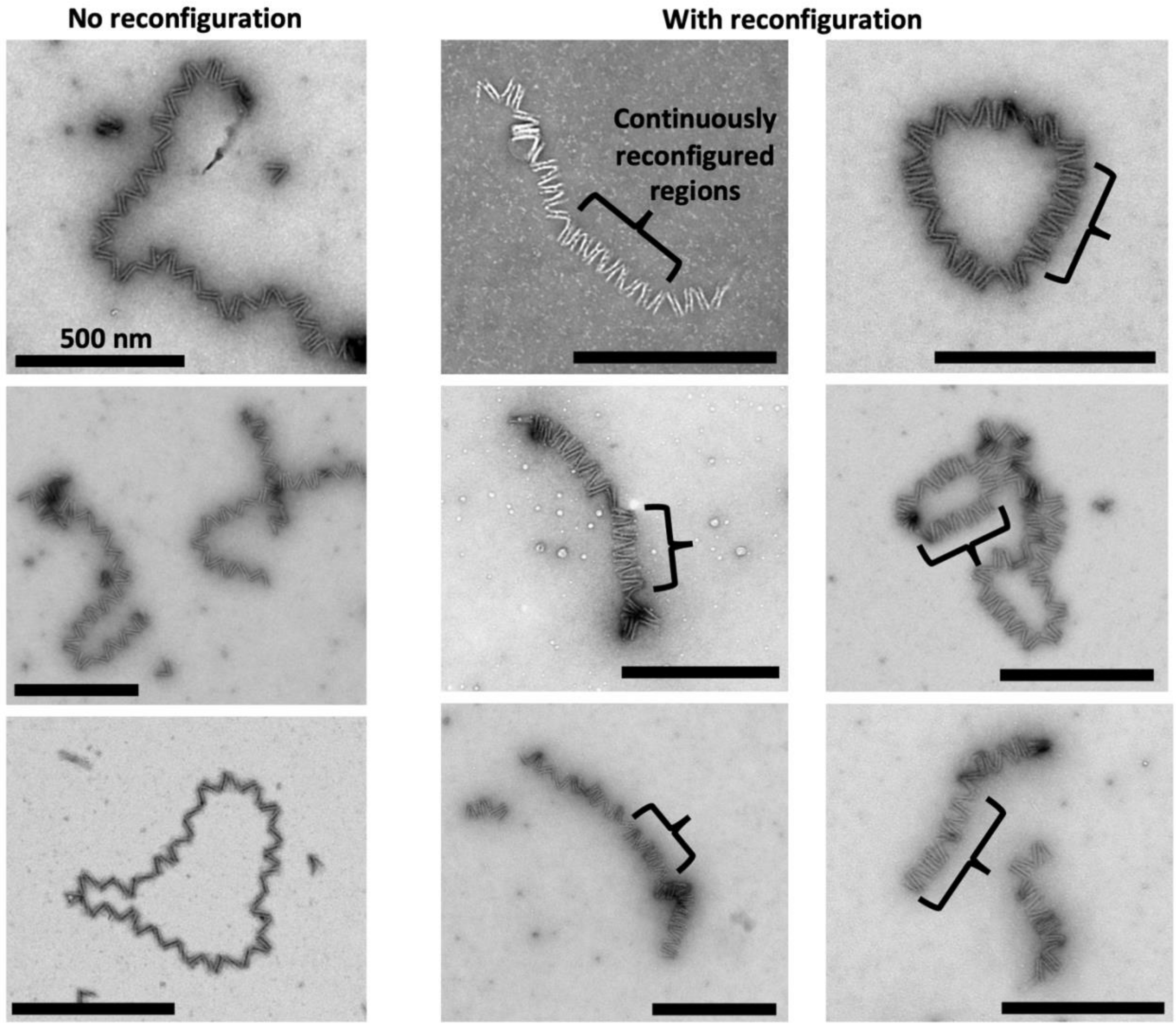

Figure S29. Example TEM images of linear hinge polymers before and after reconfiguration. All scale bars are 500nm.

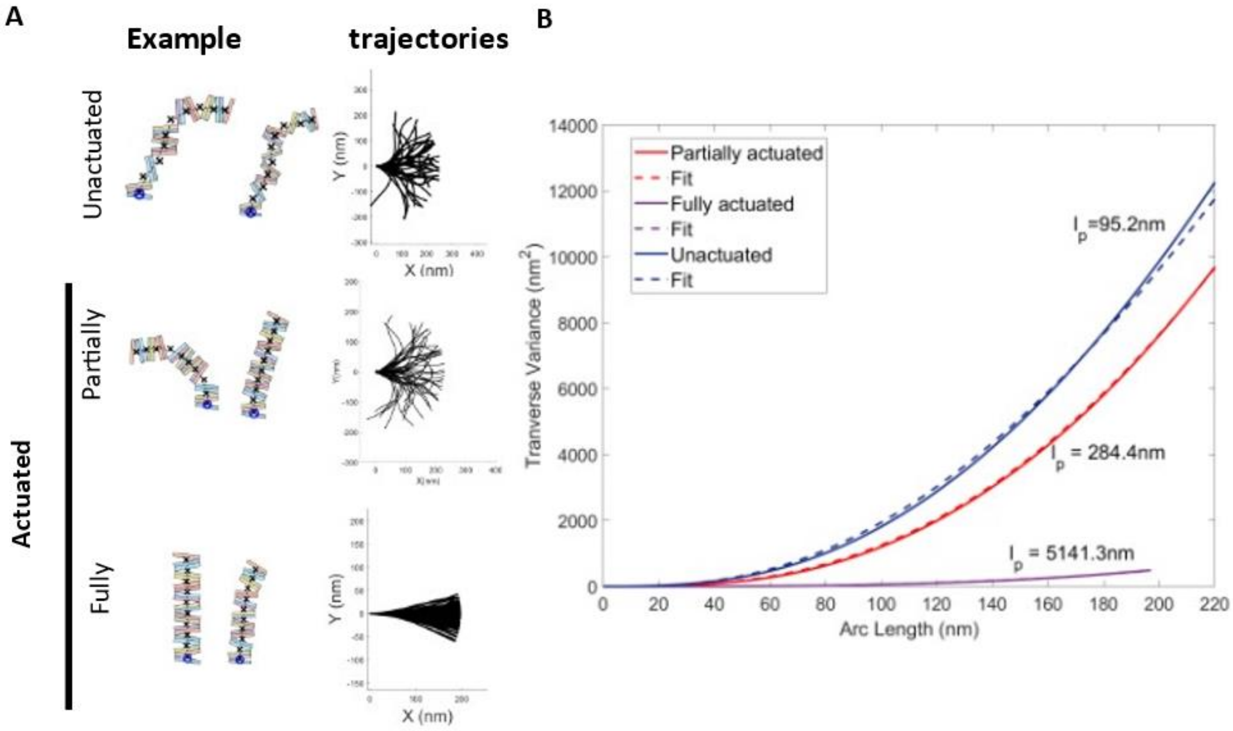

Figure S30. Persistence length measurements of simulations of unactuated, partially actuated and fully actuated linear polymers. A) Examples of 10 hinges assembled polymers with unactuated and actuated hinge. Partially actuated polymer means the simulation was built on the angle distribution of actuated polymers and fully actuated polymer means the simulation was built on the angle only picked up from angle less than 30 degrees in the same distribution. B) The transverse fluctuations were fit to equation (1) to give a persistence length.

Table S1: Oligonucleotide sequences for DNA origami design.

Element name	Sequence
Up arm main1	AAATCAAGGGCGAAAATCCTGTTTGTGAGCTAACTCACAT
Up arm main2	TCTGTCCAGGCCGATTAAAGGGATGACTCCAACGTCAAAG
Up arm main3	CAATATTAGTCACACGACCAGTAAGCCTTTAAATGAAAAA
Up arm main4	GACGCAGAACGCAACCAGCTTACGCCGGAAGATAAATCA
Up arm main5	CTAAAACGAGGGGGTAATAGTAAAATGTTTAAGGCATAGTAAGAGCAAGATTTAG
Up arm main6	CCCCAGCATTTTTTGGGTATAACGTGCTTTCCTTTGATTA
Up arm main7	AGAGGACAGATGAACGCGAGTAGTAAATTGGGGTGAATTA
Up arm main8	GTTCCGAAACCGTCTAGGGAGCTAAACAGGATCACGCAA
Up arm main9	CCATGTACCAGAGCCAAGACTTTACAAACAATGGCGGTTG
Up arm main10	AGACGACGCATTATTACAGGTAGAAAGATTCGTCAGTGC
Up arm main11	ACGCTCATGAAATGGATTATTTACTCGAGGTG
Up arm main12	CTCAGCAGAATAATTTTTTCACGTCCCTTCTGAGCCCTAAAACATCGCGAAGTATTCCACCCTC
Up arm main13	GAATACCACATTCAACAGCAAACAAGAGAATCTCATATGT
Up arm main14	CCTTATGCGATTTTAAGAGACTCCTCAAGAGAAGGGTTGA
Up arm main15	ACCCCGGTGCGCAGTCATAGTTAGCAGAAGGATCCTGATTTAAAAGAG
Up arm main16	CCTGAGTATTGCTTTGACGAGCACGGTCGAGGTGCCGTAA
Up arm main17	AACCGGATATTCAAATCGCGAAACAAAGTACATCGTCACC
Up arm main18	CACCAGAAGTGTACAGTGCTCCATGTTACTTACCACGCAT
Up arm main19	GTAATAACGCGTAAGAATACGTGGTAAAGGAAGTCACCAG
Up arm main20	ATACCAAGAGCGAGAGGCTTTTGCAAAAGAAGCGTTTACC
Up arm main21	ACAGCTGAGCTTGACGCACCACACCCGCCGCGATCCAGAA
Up arm main22	GAGTTAAAAAAGGCTCCAAAAGGATAAAAGGGCGAACCACCAGCAGAAAGCCGTCAAAATATCA
Up arm main23	TATAAGTAATCAATATTGAGAGCCAGAGGTGAATTCACCACCGCCAGC
Up arm main24	CAATTACCCAAATCAACGTAACAAATCTACGTTAATAAAA
Up arm main25	TAATTGCGAGCAACCGCAAGAATTTGCCGCCAGCAGTTGTCGA
Up arm main26	AGACTTTTAAACAACTTTCAACAGCAATATAAGCGGAATTATCATCATATTTTAAATACCGTTC
Up arm main27	TGTACATCTGCTGGTCTGGTCAGCTTGCGCTCCTGGTTTG
Up arm main28	CTGGTAATCTTAATGCGCCGCTACCCTAAAGGGAGCCCCC
Up arm main29	AAGGAACAACCACAGACAATATTTCATTGTAGC
Up arm main30	GGCGAAAAATCGGCAAAATCCCTTCATAAAGTGTAAAGCC
Up arm main31	CGAACTAAACAGTTAATGCCCCCTCCCTCAGAACCGCCAC
Up arm main32	AAAGTTAAGCAGCCTCCGGCCAGAAACGCGCGGACGGGCA
Up arm main33	CTTGAGTAAATAAGTTTTAACGGGGTCATACAACAACGCCTTATTTGA
Up arm main34	CAACAATGCGCGTGAGTTTCTTGCGAATCGAAAGACAGCATCGGCAGCGATT
Up arm main35	ATTAACCGGATGATGGCAATTCATTTTCAGCGTCCACAGA
Up arm main36	TTGCGGGAACGGAGATTTGTATCAGAGTAATCTTGACAAG

Up arm main37	GCCAACTCATCTTTGACCCCAACGAGGGTAGCAACATAGA
Up arm main38	AGGGAACCGCCACCCTCGTAACACAACTGATACCTGAAAATCACTTG
Up arm main39	AATTTCTTTGACAACAACCATCGCGCCGGAACGAGGCGCAACTTTGAA
Up arm main40	TCTAAAGCATCACTAGATACCGAAACATTCTGCGGCCTTG
Up arm main41	ATCAAACCGTTATTAATTCCTGATGTAGCATGAGTGAGAGGCTACAGAGGCTTT GAGAATACA
Up arm main42	AAACTATGCCAACAGAGATAGAATGAAAATCATAGGAAC
Up arm main43	AAGAGGCTGAACTGGCACGAGAAA
Up arm main44	GATTTAGATTGCCCTTCACCGCCTGCCAGCTGCATTAATG
Up arm main45	AGCACTAACAGCAAGCGGTCCACGACTGCCCGCTTTCCAG
Up arm main46	CCAGTCAGGACGTTGGATTATTCTGAAACATGTATCACCG
Up arm main47	CGGCGAACGTGGCGAGTTCTTTTCACCAGTGAGGGAGAGG
Up arm main48	TACAAACTTGGCTTTTAATCCTTTGCCCGAATTAAATTT
Up arm main49	CATTGCAATCACGCTGCGCGTAACGGGAAAGC
Up arm main50	CTGCTCATGCCAACGGCAGCACCGCCTAATGAGATGGTG
Up arm main51	CGGAACCTGAAGAAAAAGCTGCTCATTCAGTG
Up arm main52	GGCTGACCAATAAGGCTTGCCCTGTCATTATA
Up arm main53	GTAATCGTATCAGTTGACACTATCATAACCCTTTTT
Up arm main54	TTCATCAATCGCCTGATAAATTGTCTTGCAGG
Up arm main55	CAGCCCTCTCTGAATTAGTTTGAGTAACATTGAAAAAGA
Up arm main56	GAGAGTTGATCGGAACAGGGCGCGTACTATGGGAAGAACTC
Up arm main57	TACTCAGGGGACTTGCTGAACCTCATAGATAATACATTTGGTAAAAAA
Up arm main58	AAAATCCCAGGATTTACATTAAAACAAGCCCATCCAAAAAGGCCGCTT
Up arm main59	AATACTTCTCGTTAGAATCAGAGCTCATGAACCATCACCC
Up arm main60	AGCCGCACGAACGTGCCGGACTTGCCTGTCGTGGCCCTGA
Up arm main61	AAAGAATAAGAACGTGTTTAGACAGGAACGGTCAGTGAGGCCACCGAGGTTT GGAT
Up arm main62	CCTCAAGTGTACTGGTACAGTGCCCGTATAACGGAACAAATAAAAAC
Up arm main63	CGCTGAGGGTCGAAATCCGCGACCACCAGGCGCATAGGCT
Up arm main64	TTTAGTGATAGATTAGGATAAAACAGCAGCAATTGTATCGTATTCGGT
Up arm end 1	TTTTTTGAGGAAGGTTATTCCGGCAAACTTTTTT
Up arm end 2	TTTTTTGGAACCGAACTGACCAGACGGTCAATCATAAGTTTTTT
Up arm end 3	TTTTTTAGTACCAGGCGTGAAAGGAATTTTTTTT
Up arm end 4	TTTTTTTTCAACTTTAATCATTCTTGAGATGGTTTAATTTTTTT
Up arm end 5	CGGTTTGCGTATTGGGCGCTTTTTT
Up arm end 6	TTTTTTCAGGGTGGTTTAAAGGAAGCTGGCAAGTGTAGCGGCAGGAAAA
Up arm end 7	TTTTTTTGACGCTCAATCGTCTGGAAATACCTACATTTTTTTTT
Up arm end 8	TTTTTTATAGTTGCGCCGACAAAAACAGCTTGATACCGTTTTTT

Up arm end 9	TTTTTTGCGGTCCGTTTTTTCGTCTCGTCGCTGACGATGCT
Up arm end 10	TTTTTTGCCTGCAACAGTGCCACGCCTGGTCAGGAGCACTAACAACTAATGAAGGGT
Up arm end 11	TCAACAGTGATAAGTGCCGTCGAGAGGATTAGGATTAGCGGGGTTTTGCTCTTTTTT
Up arm end 12	AACCGATAGTTTATCAGCTTGCTTATTGGCAGGGCGGTCAGTATTAACACCTTTTTT
Up arm end 13	TTTTTTAGCGGGCGCTAGGGCGGGAAGAAAGCGAAAGGTTTTTT
Up arm start 1	GCCCGAGATAGGGTTGAGTGTTGTTCTTTTTT
Up arm start 2	TTTTTTTACGTAATGCCACTACATTAAACGGGTAAAATTTTTT
Up arm start 3	TTTTTTACACAACATACGAGGCTGGAGGTGTC
Up arm start 4	TTTTTTGTTAGTAAATGAATTTTCTTGAATAATGGAAGGGTTAG
Up arm start 5	TTTTTTATAACGCCAAAAGGAATTACGGACTGGATAAACGAAAGAGGCAAAAGGACTAA
Up arm start 6	TTTTTTCAGTTTGGAACAAGAGTCCACTATTAA
Up arm start 7	TTTTTTACAAAGAAACCACCGTAACGATTTTGCTTCATGAGG
Up arm start 8	TTTTTTCCTGAGAGTCTGGTAATGCAGATACTTTTTT
Up arm start 9	TTTTTTAAGTGTTTTTATAATACGCCAGAATCCTGAGTTTTTT
Up arm start 10	TTTTTTGATAGCTCTCACGATCATTTTGCGGATTTTTT
Up arm start 11	TTTTTTGCCAGAATGGAAATGATAATCAGAAA
Up arm start 12	AAGTTTCCGAAGGCACCAACCTAAGCGTCCAATACTGCGGAATCGTCATAAATATTCA
Up arm start 13	TATACTTCGTATGGGATCTAAAGTTTTGTCGTCTTTCCAGACTTTTTT
bottom arm main 1	AGATTCAATTATGACCAAGTTTCA
bottom arm main 2	AACCGATTTAACGGAATACCCAAATAAGAATAAATTTCATCTTCTGACTGCGCAAC
bottom arm main 3	TTCCAAGAGAACAAGCAACATAAAAACAGGGACTGAACAAAGTCAGAG
bottom arm main 4	CAAAAACAAAGGGTGAGAAAGGCCGCGAGTAACAACCCGTTCAGGAAGTACC
bottom arm main 5	TGCGCGCCAACGCCAGGGTTTTCCGCGAAAGGATCGCAAG
bottom arm main 6	TTGGGTTAGAGGGGACGCTATTACGCCAGCTGCAGTCACG
bottom arm main 7	AATTACAGAATCAAGTTTGCATTCGAGCTTCAAAGCGAACCAGAATT
bottom arm main 8	GCATTTTCGGTCATAGTCAGAGCCGCCAAACGAAAAGACC
bottom arm main 9	CAGAGGTGACCTGCAGCCAGCGGTGCACGCGTATGTAGAA
bottom arm main 10	ATGCAACTTCATTTTTCACCAGTAGCACCATTATATTGAC
bottom arm main 11	AACTTTTTGCCTCTTCGACGACAG
bottom arm main 12	GAAGGTAAACCATTAGCAAGGCCGTTAATTGCTCCTTTTG
bottom arm main 13	TAGCGATTTTCCCTTTGTGAGTGAATAACCTGTACAGCG
bottom arm main 14	ATGATATTCTGTAGCCAGCTTTCACACCGCTTGTGAATTTAATGGTTT
bottom arm main 15	CATGTTCAGGGAGGTTCGAGCGTCTTTCCAGAACGCTCAA

bottom arm main 16	TATCATATTATTATTTATCCCAATAAGGCTTA
bottom arm main 17	TGCCAGTTGTTTTAGCGAACCTCCCGACTTGCGCTAATGCAGAACGCGGTAATCAT
bottom arm main 18	CGGAATTTTACATAAACATCAAGATCAGACGACAACATAT
bottom arm main 19	CAAAGACACCACGGAATAGCGCGTTTTCATCGGAAGCCCGAAAGACTT
bottom arm main 20	GTCGCTATTCGCGTCTGGCCTTCCAACCGTTCTAGCTGAGAATTAGAGATACAT
bottom arm main 21	CAAGGCAAATAAATTAATGCCGGACGCCATCAAAAATAATTAATTAATAGCT
bottom arm main 22	ACCCGCATTGACAGGAGGTTCAAACAAATTTTTAATGGAA
bottom arm main 23	ACGTTGTATCAGACGATCCAGCGCCGTTTTCACCTTATCA
bottom arm main 24	AATATCCCAGATATAGCCAAATAAGAAACGATAATTACTA
bottom arm main 25	ATAAGAGGAAAGTACGGTGTCTGGCTGTAATACTTTTGCG
bottom arm main 26	AGAATCGCATCTTACCAACGCTAATTGAAGCC
bottom arm main 27	TTCCATATGAGTACCTGAAACGTCACCAATGACGACATTC
bottom arm main 28	GGATTAGAAACAGTTGATTCCCAAGCTAAATCGGTTGTAC
bottom arm main 29	GCCAGTGCCGGTGCGGCAAATATATAACCTCCCGCAATAAGAGGGAGG
bottom arm main 30	CAAATATCTGGTCAATAACCTGTTCAATAAATCATACAGG
bottom arm main 31	TAGAAGCAAAAGAAGTTACATACCAGTATAAAGCCAGCCTAATT
bottom arm main 32	TCTGATTACCTGTTAAGACGAGCAAACGCAATCAATAGAA
bottom arm main 33	ACTAACAAAGAAAGAAACAAGGTAATTGAGCGCTAACCTTTACA
bottom arm main 34	TCAAATCAAGCATAAATTCTGCGA
bottom arm main 35	GGAGAAGCAATGCCTGAGTAATGTACAAACGGCGGATTGAGCCAGTTTTATA
bottom arm main 36	TATGTAAACGAACAAATTCATTAAAGGTGAATTTAGAGCC
bottom arm main 37	CAAAAGGGAACCATCGATAGCAGCAAACTCCAACAGGTCA
bottom arm main 38	TACAAAGGTTAAATCAGCTCATTTGATA
bottom arm main 39	TTCAGAGGCAGGAAACAAAAAATAACGGCTTAATTG
bottom arm main 40	TTCGCAAAGCGTTTTACTTTAGCGTCAGACTGTAAGTTTA
bottom arm main 41	GGAAATTAGTTACCAGAAGGAAACTAAGAGCACGCGAGAA
bottom arm main 42	TAACGTCAACCGCGCCCAATAGCAAGCAAATCATCCTAATTTACGAGCGCCTGTTC
bottom arm main 43	CAGTAGCGCATATGGTTTACCAGCAGACTCCTTATTACGCAATACCGACCGTGTGACTGTTTAG
bottom arm main 44	TTTTTTTTAGTTAACGAATTCACAAACCGGAATCATTTTTTGTT
bottom arm main 45	GTGCATCTCCGTAATGGGATAGGTCATATATT
bottom arm main 46	GAAATTATTCATTTCATAACCAGGCAAAGCGCCATTCGCCGGATAACC
bottom arm main 47	CAGTAGGGATTCGCCTGATTGCTTAAAGGTGGTGAAAACA
bottom arm main 48	TTTTGTCATAGAAAATACATACATTGAATACCAAGATGATGAAACAAATCAATATA
bottom arm main 49	TCCGGTATTCTAAGAAAGATAAGTCCTGAACAGTTGAGGATCCCCGGG
bottom arm main 50	AGATAACCGAGTTAAGCCCAATAACGAGGAAAGGCTTAGG
bottom arm main 51	GAAAAAGCTAAATAAGGCGTTAAAAGAACTGGCATAGGTC
bottom arm main 52	TTAAATCAAGATTAGTGTCCAGAC
bottom arm main 53	ACAGGTGAGAGATAGACTTGCGGCTGGTAATGGGTCTGTGTGATAAACAA

bottom arm main 54	CCATGTTTCGTCATAAACATCCCTTCGAATTCCCTGTTTA
bottom arm main 55	TCACCGGAAGCAAATCGTTAACGGTCCTCACAAGAAAAAT
bottom arm main 56	CTATTTTTAACATCTAGCTATATTTTCATTATTAAGAG
bottom arm main 57	TAGTTTGACCATTCAAAATTAAGCAATAAAGCCTCAGCCATCAAT
bottom arm main 58	ACCAATCAGAATCATTAAAATGAAAATAGCAGTATCAGAG
bottom arm main 59	GAGAGAATAAGCCGTTTTTATTTTCATCGTAGATAATCGGCTGTCTTTCGGTCAT A
bottom arm main 60	CGCCTCCCCCCCCTTATTAGCGTTAGCAAAGCGGATTGCA
bottom arm main 61	TCAACAATCGCGAGGCACAAAATAAACAGCCAGCGTTATA
bottom arm main 62	TATCCTGACATATTTAACAACGCCAGTACCTTTTACATCGCGCC
bottom arm main 63	ACGAGTAGCCGGAAGCACCGTAAT
bottom arm main 64	ACGGGAACATTCAGGCCTAAATTTATCAAAATCATGATTAGCCAAAGA
bottom arm main 65	CAAATTCTAAATCGCGCAGAGGCGAGTATGTT
bottom arm main 66	TGAGAGACATCGCACTTGTTGGGAAGGGCGATCAAGCTTT
bottom arm main 67	TACCGAGCTACACTGGTGTGTTCAACAATCGGCGAAACTGCT
bottom arm end 1	TTTTTTTTTTAAGAAAAGTAAGATCTTACCGAAGCCCTTTTTTT
bottom arm end 2	TGAAATAGCAATAGCTCAGATAGCTGCTGATGTTTTTT
bottom arm end 3	TTTTTTTGGTGCTGCGGCCAGAATGCGGCGGGCAGTGTCAC
bottom arm end 4	TTTTTTGAATTAACTGAACACCAGCGCATTAGACGGGATTTTTT
bottom arm end 5	TTAAATGCCTTTATTTCAACGCAAGGATAAAAATTTTTTTT
bottom arm end 6	TTTTTTATAATGCTGTAGCTCAACATGTTTTAAAT
bottom arm end 7	AGCAAAATGCGGATGGCTTAGAGCTTAATTGCTGAATTTTTTT
bottom arm end 8	TTTTTTGTACCGCACTCATCGAACGGGTATTAAACCAATTTTTT
bottom arm end 9	TTTTTTTTTAGAACCCTCACGTTGGTGTTTTTTT
bottom arm end 10	TTTTTTTTAAGTTGGGTTGTGCACTCTGTTTTTT
bottom arm end 11	TTTTTTTTGAGCCATTTGGGAATATCACCGTCACCGACTTTTTT
bottom arm end 12	TTTTTTAGATGGGCGCACTGCAAGGCGATTTTTT
bottom arm start 1	TTTTTTATTTGAATTACCTTAAATCCTCATTATTTTTT
bottom arm start 2	GACGACAAAATTGTTATCCGCTCACAATTTTTTTT
bottom arm start 3	TTTTTTGGCATCAATTCTACTAATAGTAGTAGCATGAGAGATC
bottom arm start 4	TCACTGTTGCCCTTCTCCGTGGTGAATTTTTT
bottom arm start 5	CATTAAATTTTTGCTATCAGGTCATTTTTTT
bottom arm start 6	TTTTGCACATAAGAGAATATAATTTTTT
bottom arm start 7	TTTTTTCCAGAACCACCACCAGAGCCGCCGCCATCAGAGCCACCGGAAC
bottom arm start 8	TTTTTTATCACCGGAACCAGAGCCACCACCACCCTCAGAGCCGCCATTTTTT
bottom arm start 9	CTCAGAACGGAGAAACTTAATTACATTTAACAATTTCTTTTTT
bottom arm start 10	ACCCTGACTATTATAGTCAGATGCCATCTTTTCATAATCAAATTTTTT
bottom arm start 11	TTTTTTCATTTTCGAGCCAGTACCAGCTACAATTT
bottom arm start 12	TCAGATGAATATACAGTAACAACATGTAATTTAGGCAGAGGTTTTTT

bottom arm start 13	TCAAAAAGTGGGGCGCGAGCTGAAAAGGTTTTTTT
bottom arm start 14	TTTTTTAGTACCGACAAAAGGTAAAGTAATTCTTGCTA
uparm_1_1_BKI	GTTCCGAAACCGTCTAGGGAGCTATTT ATA TGG TCA ACT G
uparm_1_2_BKI	AACAGGATCACGCAAATTAACCGGATGATGGTTT ATA TGG TCA ACT G
uparm_1_3_BKI	ATCAAACCGTTATTAATTCCTGATGTAGCATGAGTGAGAGGCTACAGTTT ATA TGG TCA ACT G
uparm_2_1_BKI	CCCCAGCATTTTTTGGGTATAACGTTT ATA TGG TCA ACT G
uparm_2_2_BKI	TGCTTTCCTTTGATTAGTAATAACGCGTAAGATTT ATA TGG TCA ACT G
uparm_2_3_BKI	CAACAATGCGCGTGAGTTTCTTGCGAATCGAAAGACTTT ATA TGG TCA ACT G
uparm_3_1_BKI	GAGAGTTGATCGGAACAGGGCGCGTTT ATA TGG TCA ACT G
uparm_3_2_BKI	TACTATGGGAAGAACTCAAACTATGCCAACAGTTT ATA TGG TCA ACT G
uparm_3_3_BKI	AAAATCCCAGGATTTACATTAAAACAAGCCCATCCAAAAAGGCCGCTTTTT ATA TGG TCA ACT G
uparm_4_1_BKI	ACAGCTGAGCTTGACGCACCACACTTT ATA TGG TCA ACT G
uparm_4_2_BKI	CCGCCGCGATCCAGAACAATATTAGTCACACGTTT ATA TGG TCA ACT G
uparm_4_3_BKI	TTTAGTGATAGATTAGGATAAAACAGCAGCAATTGTATCGTATTCGGTTTT ATA TGG TCA ACT G
uparm_1_1_AEI	GTTCCGAAACCGTCTAGGGAGCTATTGT AAT ACC AGA TGG
uparm_1_2_AEI	AACAGGATCACGCAAATTAACCGGATGATGGTTGT AAT ACC AGA TGG
uparm_1_3_AEI	ATCAAACCGTTATTAATTCCTGATGTAGCATGAGTGAGAGGCTACAGTTGT AAT ACC AGA TGG
uparm_2_1_AEI	CCCCAGCATTTTTTGGGTATAACGTTGT AAT ACC AGA TGG
uparm_2_2_AEI	TGCTTTCCTTTGATTAGTAATAACGCGTAAGATTGT AAT ACC AGA TGG
uparm_2_3_AEI	CAACAATGCGCGTGAGTTTCTTGCGAATCGAAAGACTTGT AAT ACC AGA TGG
uparm_3_1_AEI	GAGAGTTGATCGGAACAGGGCGCGTTGT AAT ACC AGA TGG
uparm_3_2_AEI	TACTATGGGAAGAACTCAAACTATGCCAACAGTTGT AAT ACC AGA TGG
uparm_3_3_AEI	AAAATCCCAGGATTTACATTAAAACAAGCCCATCCAAAAAGGCCGCTTTTGT AAT ACC AGA TGG
uparm_4_1_AEI	ACAGCTGAGCTTGACGCACCACACTTGT AAT ACC AGA TGG
uparm_4_2_AEI	CCGCCGCGATCCAGAACAATATTAGTCACACGTTGT AAT ACC AGA TGG
uparm_4_3_AEI	TTTAGTGATAGATTAGGATAAAACAGCAGCAATTGTATCGTATTCGGTTTGT AAT ACC AGA TGG
bottom_1_1_BKI	CATGTTCAGGGAGGTTCGAGCGTCTTT ATA TGG TCA ACT G
bottom_1_2_BKI	TTTCCAGAACGCTCAACAGTAGGGATTCGCCTTTT ATA TGG TCA ACT G
bottom_1_3_BKI	CGGAATTTTACATAAACATCAAGATCAGACGACAACATATCAAAGACATTT ATA TGG TCA ACT G
bottom_2_1_BKI	TCAACAATCGCGAGGCACAAAATATTT ATA TGG TCA ACT G
bottom_2_2_BKI	AACAGCCAGCGTTATACAAATTCTAAATCGCGTTT ATA TGG TCA ACT G
bottom_2_3_BKI	TCTGATTACCTGTTAAGACGAGCAAACGCAATCAATTTT ATA TGG TCA ACT G
bottom_3_1_BKI	AATATCCCAGATATAGCCAAATAATTT ATA TGG TCA ACT G

bottom_3_2_BKI	GAAACGATAATTACTAGAAAAAGCTAAATAAGTTT ATA TGG TCA ACT G
bottom_3_3_BKI	ACGGGAACATTCAGGCCTAAATTTATCAAAATCATGATTAGCCAAAGATTT ATA TGG TCA ACT G
bottom_4_1_BKI	ACCAATCAGAATCATTAAAATGAATTT ATA TGG TCA ACT G
bottom_4_2_BKI	AATAGCAGTATCAGAGAGATAACCGAGTTAAGTTT ATA TGG TCA ACT G
bottom_4_3_BKI	GCCAGTGCCGGTGCGGCAAATATATAACCTCCCGCAATAAGAGGGAGGTTT ATA TGG TCA ACT G
bottom_1_1_AEI	CATGTTCAGGGAGGTTCGAGCGTCTTGT AAT ACC AGA TGG
bottom_1_2_AEI	TTTCCAGAACGCTCAACAGTAGGGATTCGCCTTTGT AAT ACC AGA TGG
bottom_1_3_AEI	CGGAATTTTACATAAACATCAAGATCAGACGACAACATATCAAAGACATTGT AAT ACC AGA TGG
bottom_2_1_AEI	TCAACAATCGCGAGGCACAAAATATTGT AAT ACC AGA TGG
bottom_2_2_AEI	AACAGCCAGCGTTATACAAATTCTAAATCGCGTTGT AAT ACC AGA TGG
bottom_2_3_AEI	TCTGATTACCTGTTAAGACGAGCAAACGCAATCAATTTGT AAT ACC AGA TGG
bottom_3_1_AEI	AATATCCCAGATATAGCCAAATAATTGT AAT ACC AGA TGG
bottom_3_2_AEI	GAAACGATAATTACTAGAAAAAGCTAAATAAGTTGT AAT ACC AGA TGG
bottom_3_3_AEI	ACGGGAACATTCAGGCCTAAATTTATCAAAATCATGATTAGCCAAAGATTGT AAT ACC AGA TGG
bottom_4_1_AEI	ACCAATCAGAATCATTAAAATGAATTGT AAT ACC AGA TGG
bottom_4_2_AEI	AATAGCAGTATCAGAGAGATAACCGAGTTAAGTTGT AAT ACC AGA TGG
bottom_4_3_AEI	GCCAGTGCCGGTGCGGCAAATATATAACCTCCCGCAATAAGAGGGAGGTTGT AAT ACC AGA TGG
Group 1 bottom1	GGTCATAGCTGTTTCAAAGGTTTCTTTGCTACCAGTCCTTGT AAT ACC AGA TGG
Group 1 bottom2	AGAATCCTTTGGCCTTTTTAACCAATAGGAAGAGGGTAGTTGT AAT ACC AGA TGG
Group 1 up1	TGGGGTGTCGGTGGTGCCATCCCAACAGCGGTTT ATA TGG TCA ACT G
Group 1 up2	TGGGGTGTCGGTGGTGCCATCCCAACAGCGGTTT ATA TGG TCA ACT G
Group 2 bottom1	CCGGGGGTTTCTGCCAGCCGGTGCCCCCTGCAAAACGACGTTGT AAT ACC AGA TGG
Group 2 bottom2	TATCGGCCCGGATTCTCCGTGGGAGTAGGTAATTGT AAT ACC AGA TGG
Group 2 up1	AATCGGCCGCACATCCTCATAACGAGGCGGCCTTT ATA TGG TCA ACT G
Group 2 up2	AACCCTCATAGCCCGGAATAGGTGAAAGTATTTTT ATA TGG TCA ACT G
Circle_actuate_overhang_up1	AAGACCCACTCAGCTTACGCCGGAAGATAAATCA
Circle_actuate_overhang_up2	AAGACCCACTACCCCGGTGCGCAGTCATAGTTAG
Circle_actuate_overhang_bot1	ACAGGTGAGAGATAGACTTGCGGCTGGCGTGCGTGTG
Circle_actuate_overhang_bot2	GAGAGATCTACAAAGGTTAAATCACGTGCGTGTG

Circle_closing strand	AGT GGA CCA GTG GGT CTT TTC ACA CGC ACG
Polymer_actuate_overhang_up1	AGTCGGGTCTATACGAAGACCCACTCGGCCAGAAACGCGCGGACGGGCA
Polymer_actuate_overhang_up2	AGTCGGGTCTATACGAAGACCCACTTATAAGTAATCAATATTGAGAGCC
Polymer_actuate_overhang_bot1	GGAGAAGCAATGCCTGAGTAATGTACAAACGGCGTGCGTGTGGACCCACTTAGGCCC
Polymer_actuate_overhang_bot2	GCCAGCTGCAGTCACGACGTTGTATCAGACGACGTGCGTGTGGACCCACTTAGGCCC
Polymer_closingstrand	AGT GGA CCA GTG GGT CTT CGT ATA GAC CCG ACT TTG GGC CTA AGT GGG TCC ACA CGC ACG

[1] S. M. Douglas, H. Dietz, T. Liedl, B. Högberg, F. Graf, W. M. Shih, *Nature* **2009**, *459*, 414–418.

[2] H. Jun, X. Wang, M. F. Parsons, W. P. Bricker, T. John, S. Li, S. Jackson, W. Chiu, M. Bathe, *Nucleic Acids Res.* **2021**, *49*, 10265–10274.

[3] E. Stahl, T. G. Martin, F. Praetorius, H. Dietz, *Angew. Chem. Int. Ed Engl.* **2014**, *53*, 12735–12740.

[4] "peakfit.m," can be found under https://www.mathworks.com/matlabcentral/fileexchange/23611-peakfit-m, **2023**.

[5] A. Buchberger, C. R. Simmons, N. E. Fahmi, R. Freeman, N. Stephanopoulos, *J. Am. Chem. Soc.* **2020**, *142*, 1406–1416.
